# A Novel TRIM9 Protein Promotes NF-κB Activation Through Interacting With LvIMD in Shrimp During WSSV Infection

**DOI:** 10.3389/fimmu.2022.819881

**Published:** 2022-02-23

**Authors:** Mingzhe Sun, Shihao Li, Songjun Jin, Xuechun Li, Jianhai Xiang, Fuhua Li

**Affiliations:** ^1^ Chinese Academy of Sciences (CAS) and Shandong Province Key Laboratory of Experimental Marine Biology, Institute of Oceanology, Chinese Academy of Sciences, Qingdao, China; ^2^ Laboratory for Marine Biology and Biotechnology, Qingdao National Laboratory for Marine Science and Technology, Qingdao, China; ^3^ Center for Ocean Mega-Science, Chinese Academy of Sciences, Qingdao, China; ^4^ University of Chinese Academy of Sciences, Beijing, China; ^5^ The Innovation of Seed Design, Chinese Academy of Sciences, Wuhan, China

**Keywords:** TRIM9, lymphoid organ, IMD signaling pathway, WSSV, *Litopenaeus vannamei*

## Abstract

The TRIpartite Motif (TRIM) proteins play key roles in cell differentiation, apoptosis, development, autophagy, and innate immunity in vertebrates. In the present study, a novel TRIM9 homolog (designated as *LvTRIM9-1*) specifically expressed in the lymphoid organ of shrimp was identified from the Pacific whiteleg shrimp *Litopenaeus vannamei*. Its deduced amino acid sequence possesses the typical features of TRIM proteins, including a RING domain, two B-boxes, a coiled-coil domain, a FN3 domain, and a SPRY domain. The transcripts of *LvTRIM9-1* were mainly located in the lymphoid tubules of the lymphoid organ. Knockdown of *LvTRIM9-1* could apparently inhibit the transcriptions of some genes from white spot syndrome virus (WSSV) and reduce the viral propagation in the lymphoid organ. Overexpression of *LvTRIM9-1* in mammalian cells could activate the promoter activity of NF-κB, and an *in vivo* experiment in shrimp showed that knockdown of *LvTRIM9-1* reduced the expression of *LvRelish* in the lymphoid organ. Yeast two-hybridization and co-immunoprecipitation (Co-IP) assays confirmed that LvTRIM9-1 could directly interact with LvIMD, a key component of the IMD pathway, through its SPRY domain. These data suggest that LvTRIM9-1 could activate the IMD pathway in shrimp *via* interaction with LvIMD. This is the first evidence to show the regulation of a TRIM9 protein on the IMD pathway through its direct interaction with IMD, which will enrich our knowledge on the role of TRIM proteins in innate immunity of invertebrates.

## Introduction

White spot syndrome virus (WSSV), the causative agent of white spot syndrome (WSS), could cause 100% mortality within 7–10 days and lead to serious economic loss in shrimp aquaculture worldwide (1, 2). Several signaling pathways including Toll pathway, IMD pathway, and JAK-STAT pathway are reported to be involved in the immunity of shrimp during WSSV infection (3). The Toll and IMD pathways, two NF-κB signaling pathways mediated by the transcriptional factor Dorsal and Relish, can regulate the expression of various antimicrobial peptides (AMPs) that directly kill foreign pathogens. However, these two pathways could also be subverted and hijacked by the virus to favor its propagation in shrimp during WSSV infection (1). Activation of Toll or IMD pathway promotes the expression of several WSSV immediate early genes, like *wsv069* and *wsv303* (4, 5). Therefore, activation of these two pathways might work as a double-edged sword in shrimp during WSSV infection.

The shrimp IMD pathway mediates a signaling cascade with high similarity to the *Drosophila* IMD pathway (6). The *Drosophila* IMD pathway is triggered by peptidoglycan *via* its receptor peptidoglycan recognition protein (PGRP) and leads to the recruitment of a signaling complex consisting of IMD, Fas-associated protein with a death domain (FADD), and the caspase-8 homolog death-related ced-3/Nedd2-like protein (DREDD) (7). IMD is ubiquitinated and then functions as a scaffold for interaction with the TAK1/TAB2 complex and consequently activates the IKK/Relish branch to initiate the transcription of target genes (8). Although some homologs of the *Drosophila* IMD pathway including PGRP, FADD, and DREDD have not yet been identified in shrimp, some core components, such as IMD, TAB2, TAB1, TAK1, IKKβ, IAP2, and the transcription factor Relish, have been reported in shrimp, and interplay among these core components can regulate the expression of several AMPs in insect cells and *in vivo* (9–13). Shrimp IMD encodes a death-domain-containing protein and could induce the expression of AMP genes in S2 cells (6). Shrimp Relish is activated *via* phosphorylation and cleavage to release its N-terminal Rel homology domain (RHD), which is then translocated into the nucleus to trigger the expression of several AMPs (9).

The immune function of the IMD pathway in shrimp is regulated by multiple molecules, such as NF-κB repressing factor, Akirin, and TRIpartite Motif (TRIM) proteins (1). TRIM proteins, one family of E3 ubiquitin ligases, could activate or inhibit NF-κB signal transduction through mediating various types of ubiquitination in vertebrates (14). In mammals, TRIM proteins are grouped into eleven classes according to their diverse C-terminal domains and exhibit different functions in different ways (15, 16). For example, TRIM8, TRIM20, TRIM23, and TRIM25 exhibit positive regulatory effects on the NF-κB pathway, while TRIM9, TRIM19, TRIM21, TRIM27, and TRIM30α show negative regulatory functions (14, 17). In humans, two isoforms of TRIM9, generated by alternative splicing of one coding sequence, negatively regulate the NF-κB pathway *via* interacting with β-transducin repeat-containing protein (β-TrCP) (18, 19). These data suggest that TRIM proteins play important roles in regulating NF-κB pathways. In crustaceans, different TRIM proteins have been identified with important functions during pathogen infection, since our first identification of a TRIM9 in *Litopenaeus vannamei* (20). TRIM50 can inhibit WSSV propagation through ubiquitinating viral envelope proteins and leading them to degradation by autophagy in *Penaeus monodon* (21). TRIM32 could inhibit WSSV propagation in *Cherax quadricarinatus* and *L. vannamei*, but the mechanism is unclear (22, 23). On the contrary, TRIM23 could promote WSSV infection in *Macrobrachium nipponense* and TRIM9 could promote *Vibrio parahaemolyticus* infection in *P. monodon*, both through a negative regulation on the expression of antimicrobial peptides (24, 25). However, the functional mechanism of TRIM proteins during pathogens infection in crustaceans is still largely unclear.

TRIM9 is a typical class-I TRIM protein and coexists with other five paralogs in mammals, but TRIM9 is generally considered as a unique protein with the class-I motif architecture in invertebrate (16, 26, 27). In our previous study, an E3 ubiquitin ligase TRIM9 was identified in the Pacific whiteleg shrimp *L. vannamei*, and it could interact with Lvβ-TrCP to regulate the NF-κB pathway to accelerate WSSV propagation in shrimp (20). In the present study, a novel TRIM9 (designated as *LvTRIM9-1*) highly expressed in the lymphoid organ of *L. vannamei* was identified. Functional analyses showed that *LvTRIM9-1* could accelerate WSSV propagation in shrimp through a different mechanism in regulating the NF-κB pathway during WSSV infection. The present study will provide important information to understand the roles of TRIM proteins in the immunity of crustaceans.

## Materials and Methods

### Experimental Animals and Viral Challenge

Healthy adult shrimp cultured in our lab, with a body weight of 19.4 ± 1.1 g, were used for tissue distribution analysis and WSSV challenge experiments. Before experiments, shrimp were reared in air-pumped circulating seawater at 25 ± 1°C and fed with commercial food pellet for about a week. Shrimp were selected randomly in each experiment. Hemolymph was collected from the ventral sinus located at the first abdominal segment using a syringe with equal volume of precooled anticoagulant solution (115 mmol l^−1^ glucose, 27 mmol l^−1^ sodium citrate, 336 mmol l^−1^ NaCl, 9 mmol l^−1^ EDTA•Na_2_•2H_2_O, pH 7.4) (28). Hemocytes were immediately collected by centrifugation at 1,000 g, 4°C, for 5 min. Different tissues including lymphoid organ, hepatopancreas, gills, intestine, epidermis, muscle, stomach, and heart were dissected from 9 individuals, and those from three individuals were put together as one sample. The samples were preserved in liquid nitrogen for tissue distribution analysis.

Lymphoid organs dissected from shrimp were fixed separately in RNA friendly fixative (RFF) (29) for 48 h at 4°C. After being dehydrated with serial ethanol and cleared with serial xylene, they were embedded in paraffin (Sigma, San Francisco, CA, USA). Histological sections with thickness of 5–7 μm were used for hematoxylin and eosin (H&E) staining and *in situ* hybridization analysis.

To examine the *LvTRIM9-1* expression pattern after WSSV challenge, 90 individuals were randomly divided into two groups. For WSSV challenge, the virus particles were prepared according to the method described by Sun et al. (30). The WSSV particles were diluted in sterilized phosphate-buffered saline (PBS) at a final concentration of 800 copies µl^-1^, and 10 μl was injected into each shrimp at the III and IV abdominal segments in the WSSV group. The equal volume of PBS was injected into each shrimp in the PBS group. At 0, 3, 6, 12, and 24 h post WSSV infection (hpi), the lymphoid organs of 9 individuals in each group were collected for quantifying the mRNA expression levels, and those from three individuals were put together as one sample.

### Cloning and Sequence Analysis of the LvTRIM9-1 Gene

The total RNA was extracted by TRIzol reagent (Takara, Tokyo, Japan), and the cDNA template was synthesized using RevertAid First Strand cDNA Synthesis Kit (Thermo Fisher Scientific, Waltham, MA, USA) according to the manufacturer’s protocols. Two specific primers LvTRIM9-1-1F and LvTRIM9-1-1R (**Table S1**) were designed to amplify and validate the sequence of *LvTRIM9-1* from the genome and transcriptome data (31). PrimeStar GXL DNA Polymerase (Takara, Japan) was used to amplify the gene. After the quality was assessed by electrophoresis on 1% agarose gel, the specific product was purified using Gel Extraction Kit (Omega, Norcross, GA, USA), cloned into the pMD19-T vector (TaKaRa, Japan), and transformed into DH5α competent cells (TransGen, China) for sequencing.

The complete ORF and amino acid sequence of LvTRIM9-1 was deduced using ORFfinder (https://www.ncbi.nlm.nih.gov/orffinder/). Conserved protein domains were predicted with SMART (http://smart.embl-heidelberg.de/). Different TRIM protein sequences (**Table S2**) were obtained from the UniProtKB/Swiss-prot and NCBI database. Multiple-sequence alignment and phylogenic analysis were performed using the neighbor-joining (NJ) method by ClustalW and MEGA 6.

### Quantitative Real-Time qPCR and RT-PCR

The expression of mRNA was examined by using quantitative real-time PCR (qPCR) as previously described (20). In brief, after extraction of total RNA using the TRIzol reagent (Takara, Japan), the cDNA template was synthesized using the PrimeScript RT Reagent Kit (Takara, Japan) with random primers, and qPCR was performed using THUNDERBIRD™ SYBR^®^ qPCR Mix (Toyobo, Osaka, Japan) to quantify the mRNA expression levels. The primers used for qPCR analysis are listed in **Table S1**. The *18S rRNA* (GenBank No. EU920969) was employed as an internal control for cDNA normalization. The PCR product was denatured to produce a melting curve to check the specificity of the PCR product.

RT-PCR was conducted to analyze the distribution of *LvTRIM9-1* among different shrimp tissues and its expression level in HEK293T cells. The amount of cDNA templates from shrimp tissues was quantified using *18S rRNA* as an internal reference gene following the PCR condition described below: denaturation at 94°C for 2 min; 16 cycles of 94°C for 20 s, 56°C for 20 s, and 72°C for 20 s. The amount of cDNA templates from HEK293 cells was quantified using *HsActin* (GenBank No. NM_001101.5) as an internal reference gene following the PCR condition described below: denaturation at 94°C for 2 min; 26 cycles of 94°C for 20 s, 56°C for 20 s, and 72°C for 20 s. An equal amount of cDNA from different tissues or HEK293T cells was used for detecting the expression pattern of *LvTRIM9-1* transcripts following the PCR condition described below: denaturation at 94°C for 2 min; 36 cycles of 94°C for 20 s, 61°C for 20 s, and 72°C for 30 s. The primers are listed in **Table S1**. The PCR products were detected by electrophoresis on 2% agarose gel.

### *In Situ* Hybridization

Primers LvTRIM9-1-pF1 with the T7 promoter and LvTRIM9-1-pR1 were designed to amplify a 551-bp fragment of LvTRIM9-1 as the template for sense probe synthesis. Primers LvTRIM9-1-pR2 with the T7 promoter and LvTRIM9-1-pF2 were designed for the template for antisense probe synthesis (**Table S1**). The PCR products were purified by MiniBEST DNA Fragment Purification Kit (Takara, Japan) and assessed by electrophoresis on 1.5% agarose gel. Digoxygenin (DIG)-labeled riboprobes were synthesized through *in vitro* transcription using DIG RNA Labeling Mixture (Roche, Mannheim, Germany) and TranscriptAid T7 High Yield Transcription Kit (Thermo Fisher Scientific, USA). After assessing the concentration and quality of synthesized RNA probes by NanoDrop 2000 (Thermo Fisher Scientific, USA) and agarose electrophoresis, the DIG-labeled RNA probes were stored at -80°C for further use.

The paraffin-embedded lymphoid organ was sectioned into slices of 5–7 μm. Hybridization was performed following general protocol of the DIG RNA Labeling Kit (Roche, Germany). The concentration of both sense RNA probe and antisense RNA probe was 1 ng μl^-1^. The signals were visualized by the color reaction using NBT/BCIP Stock Solution (Roche, Germany) and observed through a Nikon Eclipse 80i microscope (Nikon, Tokyo, Japan).

### RNA Interference, DNA Extraction, and WSSV Load Quantification

A pair of primers with T7 promoter sequence, LvTRIM9-1-dsF and LvTRIM9-1-dsR (**Table S1**), was designed to amplify a 551-bp fragment of the *LvTRIM9-1* gene. Primers of EGFP-dsF and EGFP-dsR with the T7 promoter sequence (**Table S1**) were used to clone a 289-bp DNA fragment of enhanced green fluorescent protein (EGFP) gene based on pEGFP-N1 plasmid for dsRNA synthesis. The method for synthesis and purification of dsRNA was the same as described previously (32). Briefly, the PCR products were assessed by electrophoresis on 1% agarose gel and purified using MiniBEST Fragment Purification Kit (Takara, Japan). The purified products were used to synthesize the corresponding dsRNA using TranscriptAid T7 High Yield Transcription Kit (Thermo Fisher Scientific, USA). Redundant single-strand RNA was digested by RNaseA (Takara, Japan). The concentration and quality of synthesized dsRNA were assessed by NanoDrop 2000 (Thermo Fisher Scientific, USA) and electrophoresis on 1% agarose gel, respectively. All the purified dsRNA was stored at -80°C for further experiment.

To optimize the silencing efficiency of *LvTRIM9-1* dsRNA, healthy shrimp with an average body weight of 12.3 g were divided into two groups, dsTRIM9-1 (injected with *LvTRIM9-1* dsRNA) and dsEGFP (injected with *EGFP* dsRNA). Different dosages of dsRNA including 0.05, 0.10, and 0.20 µg/g were injected into each shrimp. After detecting the transcription level of *LvTRIM9-1* at 48 h postinjection, the dosage of 0.05 µg dsRNA per gram body weight was selected for further RNAi experiments. A total of 90 individuals were randomly divided into two groups including dsEGFP group and dsTRIM9-1 group. Specific dsRNA for *EGFP* and *LvTRIM9-1* genes with the dose of 0.05 μg/g were injected into the last abdominal segment of each shrimp separately. After 48 h, each shrimp in different treatments was injected with 8,000 copies WSSV. To assess the effect of LvTRIM9-1 on the WSSV propagation among different target tissues, the lymphoid organ and epidermis from 15 individuals in each group were collected at 0, 24, and 48 h post virus infection for RNA and DNA extraction, and the same tissues from 5 individuals were put together as one sample.

DNA was extracted from lymphoid organs and epidermis using the Genomic DNA Kit (Tiangen, Beijing, China) according to the manufacturer’s instructions. Protease K (Roche, Germany) was added additionally at a final concentration of 5.7 mg/ml for digestion. Extracted DNA was quantified by NanoDrop 2000 (Thermo Fisher Scientific, USA). Viral loads in the lymphoid organs and epidermis were quantitatively analyzed using SYBR Green-based qPCR according to the method described by Sun et al. (30). Briefly, the DNA encoding the extracellular part of the WSSV envelope protein VP28 was amplified and cloned into a pMD19-T simple vector (Takara, Japan). The purified and quantified plasmid was used to generate a standard curve. The DNA of the lymphoid organs and epidermis were used to detect the viral loads with primers VP28-qF and VP28-qR (**Table S1**). Each assay was carried out in quadruplicate.

### Plasmid Construction, Cell Culture, and Transfection

In dual-luciferase reporter assays, the open reading frame (ORF) of LvTRIM9-1 was amplified using PrimeSTAR^®^ GXL DNA Polymerase (Takara, Japan) with specific primers (**Table S1**) and inserted into the pCDNA3.1 vector using In-Fusion HD Cloning Plus (Clontech, Mountain View, CA, USA). The plasmids of NF-κB reporter genes were purchased from Beyotime Biotechnology Corporation of China (Shanghai), and pRL-TK Renilla luciferase plasmids were purchased from Promega Company of USA (Madison, WI). In Y2H assays, the primers were designed based on the sequence of LvTAK1 (GenBank No. KU522004), LvTRAF6 (GenBank No. HM581680), LvIMD (GenBank No. FJ592176), LvGSK3β (GenBank No. KU641425), and Lvβ-TrCP (GenBank No. XM_027360659). The ORFs of LvTAK1, LvTRAF6, LvIMD, LvGSK3β, and Lvβ-TrCP were amplified by these primers and then cloned into the pGADT7 vector (Clontech, USA). They were designated as pGAD-TAK1, pGAD-TRAF6, pGAD-IMD, pGAD-GSK3β, or pGAD-β-TrCP and used as prey plasmid. The ORF of LvTRIM9-1 was amplified by primers pGBK-TRIM9-1-F/R and then cloned into the pGBKT7 vector (Clontech, USA), which was designated as pGBK-TRIM9-1 and used as bait plasmid. In Co-IP assays, the ORF and truncated mutants of LvTRIM9-1 and ORF of LvIMD were amplified by primers as listed in **Table S1** and inserted into pDHsp70-Flag/His and pDHsp70-V5/His vectors [generously provided by Pro Lo (33)] for Flag or V5 fusion protein expression.

Sf9 cells were purchased from the China Center for Type Culture Collection (Wuhan, China) and cultured in Sf-900™ II SFM (Gibco, Grand Island, NY, USA) at 27°C and subcultured every 3–4 days. Plasmids were transfected into Sf9 cells using Lipofectamine 3000 reagent (Life Technologies, Carlsbad, CA, USA) according to the manufacturer’s instructions. HEK293T cells were cultured in Dulbecco’s modified Eagle’s medium (high glucose) (Gibco, USA) supplemented with 10% heat-inactivated fetal bovine serum (Gibco, USA) and 1× penicillin–streptomycin solution (Solarbio, Beijing, China). Cells were grown in an atmosphere of 95% air/5% CO_2_ at 37°C and subcultured every 2 days. Plasmids were transfected into HEK293T cells using Xfect Transfection Reagent (Takara, Japan) according to the manufacturer’s instructions.

### Dual-Luciferase Reporter Assays

Dual-luciferase reporter assays were performed in HEK293T cells to detect the regulatory effect of LvTRIM9-1 protein on the NF-κB pathway. Briefly, cells in 24-well plates (Corning, Tewksbury, MA, USA) were transfected with 0.1 μg of NF-κB reporter gene plasmid, 0.05 μg of pRL-TK Renilla luciferase plasmid (Promega), and varying amounts of expression plasmids or empty expression vector (as a control). The pRL-TK Renilla luciferase plasmid was used as an internal control. At 24 h post-transfection, the Dual Luciferase Reporter Assay Kit (Vazyme Biotech, Nanjing, China) was used to measure the activities of firefly and Renilla luciferases according to the manufacturer’s instructions. The level of *LvTRIM9-1* mRNA and protein were detected by RT-PCR using primers for amplification of *HsActin* and *LvTRIM9-1* (**Table S1**) and Western blotting using anti-His antibody (Cell Signaling Technology, Danvers, MA, USA). Experiments were performed in triplicate.

### Yeast Two-Hybrid Assay

In order to know the antiviral signaling pathway regulated by LvTRIM9-1, a yeast two-hybrid system was used to detect the interaction between the LvTRIM9-1 and candidate genes of the NF-κB pathway. The prey plasmids pGAD-TAK1, pGAD-TRAF6, pGAD-IMD, pGAD-GSK3β, or pGAD-β-TrCP were co-transformed into yeast strain Y2H Gold with the bait plasmid pGBK-TRIM9-1 by the lithium acetate transformation procedure according to the Matchmaker protocol manual (Clontech, USA). In addition, pGAD-TAK1, pGAD-TRAF6, pGAD-IMD, pGAD-GSK3β, or pGAD-β-TrCP co-transformed with pGBKT7 and pGBK-TRIM9-1 co-transformed with pGADT7 was used to detect autoactivation. The pGBK-p53 and pGAD-T-antigens were used for positive control. The pGBK-Lam and pGAD-T-antigens were used for negative control. After co-transforming, the yeast transformants were coated on SD/-Leu/-Trp (DDO) plates, growing 3 to 5 days at 30°C. All clones growing on DDO were collected and cultured on SD/-Leu/-Trp/X-a-gal/AbA (DDO/X/A) for primary screening. After 5 to 7 days culture at 30°C, colonies were selected and plated onto SD/-Ade/-His/-Leu/-Trp/X-α-Gal/AbA (QDO/X/A) plates to perform β-galactosidase activity analysis.

### Co-Immunoprecipitation

Co-immunoprecipitation assays were performed in Sf9 cells to detect the critical domain of LvTRIM9-1 protein for its interaction with LvIMD. The cells were transfected with different plasmids (pDHsp-V5-LvTRIM9-1, pDHsp-V5-LvTRIM9-1-ΔC1, pDHsp-V5-LvTRIM9-1-ΔN6, pDHsp-V5-LvIMD, pDHsp-V5, pDHsp-Flag-LvTRIM9-1, pDHsp-Flag-LvIMD, pDHsp-Flag) and cultured overnight at 27°C. These cells were subjected to heat-shock treatment (42°C for 30 min) and subsequently cooled to 27°C. The cells are harvested in cell lysis buffer (Beyotime, China) 24 h after the heat shock treatment. Input samples were prepared from the cell lysate, and the remaining lysates were mixed with anti-Flag M2 magnetic beads (Sigma, USA) under gentle shaking on a roller at 4°C for 2 h. The beads were then washed two times with PBS buffer and cell lysis buffer. Input and Co-IP samples were incubated with 4× LDS sample buffer (GenScript, Nanjing, China) at 100°C for 10 min. Proteins were analyzed by Western blotting using anti-V5 and anti-Flag antibodies (Cell Signaling Technology, USA).

### Statistical Analysis

All assays described above were biologically repeated for three times. For quantitative real-time PCR, four replicates were set for each sample. The relative transcription levels of different genes detected in present study were obtained using the 2^-ΔΔCt^ method (34), and the WSSV copy number per nanogram DNA was obtained according to the standard curve. The numerical data from each experiment were analyzed to calculate the mean and standard deviation of triplicate assays. The significant differences among groups were subjected to one-way analysis of variance (one-way ANOVA) and multiple comparisons by using the SPSS 19.0 program. Statistically significant difference was set at *p* < 0.05 and extremely significant difference at *p* < 0.01.

## Results

### LvTRIM9-1 Is a Novel TRIM9 Protein With Tissue-Specific Expression Pattern

**Figure 1:**
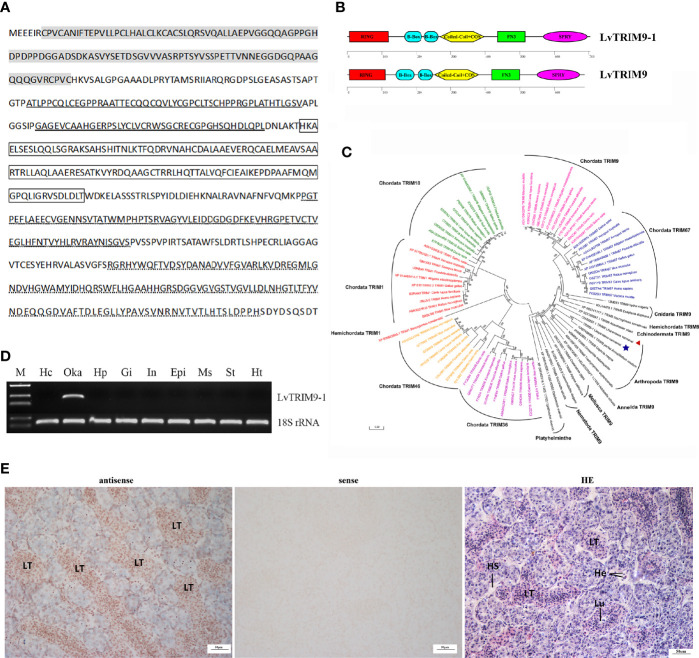
Sequence information and tissue expression pattern of *LvTRIM9-1*. **(A)** Deduced amino acid sequence of LvTRIM9-1. The RING domain was marked in gray. B-Box domains were bolded underlined. Coiled-coil and COS domains were marked in box. The FN3 domain was waved underlined. The SPRY domain was dotted underlined. **(B)** Schematic diagrams of LvTRIM9-1 and LvTRIM9. The RING, B-box, Coiled-coil, COS, FN3, and SPRY domains are illustrated. **(C)** Phylogenetic analysis of Class I TRIM proteins across animal phyla based on protein sequences. Different TRIM proteins were classified into seven branches shown with different colors (green, Chordata TRIM18; red, Chordata and Hemichordata TRIM1; yellow, Chordata TRIM46; purple, Chordata TRIM36; blue, Chordata TRIM67; pink, Chordata TRIM9; black, TRIM9 in non-vertebrate animal phyla). LvTRIM9 was marked with a red triangle, and LvTRIM9-1 was marked with a blue five-pointed star. The TRIMs were shown with UniprotKB/Swiss-Prot or GenBank accession numbers listed in **Table S2**. The neighbor-joining phylogenetic tree was built by MEGA 6, with bootstrap of 1000. **(D)** Expression patterns of *LvTRIM9-1* in different tissues of *L. vannamei*. The 18S rRNA gene was used as the internal reference. Hc, hemocytes; Oka, lymphoid organ; Hp, hepatopancreas; Gi, gill; In, intestine; Epi, epidermis; Ms, muscle; St, stomach; Ht, heart. **(E)** Localization of *LvTRIM9-1* transcripts in lymphoid organ of *L. vannamei*. Hematoxylin–eosin (H&E) staining (HE) and sense probe (sense) were used as control of the antisense probe hybridization (antisense).

The transcript of *LvTRIM9-1* obtained from the transcriptome database of *L. vannamei* was validated by PCR and confirmed by sequencing. The length of the ORF of LvTRIM9-1 was 2112 base pairs (bp), encoding 703 amino acids (aa). The deduced amino acid sequence of LvTRIM9-1 contained all conserved domains of TRIM proteins, including the RING domain (Cys^7^-Cys^119^), two B-Box-type zinc finger domains (Ala^167^-Val^216^, Gly^225^-Leu^264^), and one coiled-coil domain and COS domain (His^271^-Thr^397^), followed by one fibronectin type III repeat (FNIII) (Pro^437^-Ser^515^) and one SPRY domain (Arg^569^-His^694^) at the C terminus (**Figures 1A, B** **)**, like those domains of vertebrate TRIM1, TRIM9, and TRIM67. Although LvTRIM9-1 and LvTRIM9 shared the same functional domains (**Figure 1B**), the similarity between their amino acid sequences was around 41.87%. Phylogenetic analysis showed that LvTRIM9-1 was firstly clustered with Arthropoda TRIM9 proteins including LvTRIM9 and then clustered with TRIM9 proteins from other invertebrates. In addition, Chordata TRIM1, TRIM18, TRIM9, TRIM67, TRIM36, and TRIM46 were all clustered into separated branches (**Figure 1C**). These data indicated that LvTRIM9-1 protein was encoded by a new TRIM9 gene in *L. vannamei*.

Tissue distribution analysis showed that the transcripts of *LvTRIM9-1* were specifically expressed in lymphoid organ (**Figure 1D**). *In situ* hybridization analysis showed that *LvTRIM9-1* transcripts were mainly located in the lymphoid tubules (LT) of lymphoid organ (**Figure 1E**).

### LvTRIM9-1 Benefits WSSV Propagation in Lymphoid Organ

**Figure 2:**
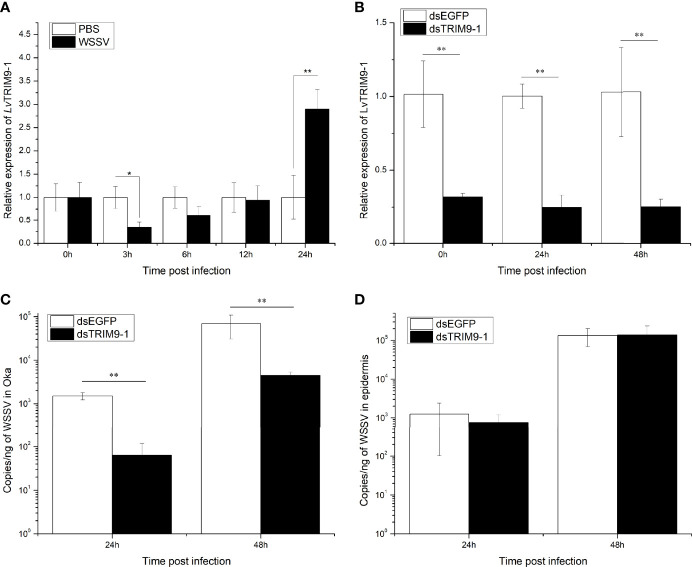
LvTRIM9-1 is beneficial for WSSV infection in lymphoid organ. **(A)** Expression levels of *LvTRIM9-1* in the lymphoid organ of shrimp at different time post-WSSV challenge. PBS stands for PBS injection group, and WSSV stands for WSSV injection group. **(B)** Inhibition efficiency of LvTRIM9-1 dsRNA. Expression levels of *LvTRIM9-1* in the lymphoid organ of LvTRIM9-1 silencing and control shrimp after 0, 24, and 48 h post WSSV infection. **(C, D)** Amount of WSSV particles in lymphoid organs and epidermis of shrimp at different hours after silencing of *LvTRIM9-1* and WSSV infection. dsEGFP, injected with *EGFP* dsRNA and WSSV; dsTRIM9-1, injected with *LvTRIM9-1* dsRNA and WSSV. Stars (*) indicate significant difference (*p* < 0.05) and (**) indicate extremely significant difference (*p* < 0.01) of the gene expression levels between the two treatments. All assays described above were biologically repeated for three times.

Since *LvTRIM9-1* showed a specific expression pattern in lymphoid organ, its expression profiles in lymphoid organ after virus infection were detected by qPCR (**Figure 2A**). After WSSV infection, the expression level of *LvTRIM9-1* in lymphoid organ decreased slightly at 3 h post WSSV infection (hpi) and increased significantly at 24 hpi, which was 2.90-fold of that in PBS group (*p* < 0.01).

RNAi and subsequent virus infection were performed to study the immunological function of LvTRIM9-1. After dosage optimization, 0.05 μg dsRNA per gram of body weight was used to knock down the expression of *LvTRIM9-1* by 68.0% (**Figure 2B**). The WSSV copy numbers in lymphoid organ and epidermis of shrimp from dsEGFP and dsTRIM9-1 groups were detected at 24 and 48 hpi to assess the viral propagation after *LvTRIM9-1* silencing. The viral copy number in lymphoid organ from the dsEGFP group were about 1.51 × 10^3^ and 6.96 × 10^4^ copies ng^-1^ DNA at 24 and at 48 hpi, while they were about 6.38 × 10^1^ and 4.45 × 10^3^ copies ng^-1^ DNA at 24 and 48 hpi in shrimp from the dsTRIM9-1 group, which were much lower (*p* < 0.01) than those for the dsEGFP group (**Figure 2C**). However, the viral number in epidermis showed no significant difference between dsEGFP and dsTRIM9-1 groups (**Figure 2D**).

### LvTRIM9-1 Promotes Viral Propagation *via* Activating the NF-κB Pathway in Lymphoid Organ

**Figure 3:**
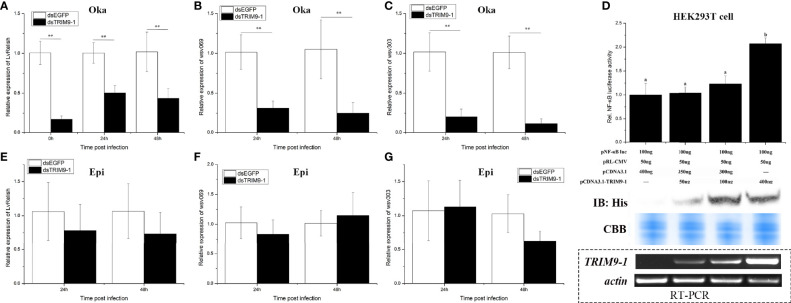
LvTRIM9-1 enhanced WSSV transcription through activation of the NF-κB pathway. **(A–C)** The relative expression level of *LvRelish*, *wsv069*, and *wsv303* in lymphoid organ at different hours after silencing of *LvTRIM9-1* and WSSV infection. **(D)** LvTRIM9-1 activated the NF-κB promoter activity in a concentration-dependent manner. Activation was detected using dual luciferase reporter assays in human HEK293T cells. Results are displayed as the fold change from the control group. Numbers (a, b) indicate extremely significant difference (*p* < 0.01) of relative luciferase activities among different groups. The expression of *LvTRIM9-1* was detected by Western blotting using the anti-His antibody and RT-PCR. Blots were stained with Coomassie brilliant blue (CBB) to verify equal loading. **(E–G)** The relative expression level of *LvRelish*, *wsv069*, and *wsv303* in epidermis at different hours after silencing of *LvTRIM9-1* and WSSV infection. dsEGFP, injected with *EGFP* dsRNA and WSSV; dsTRIM9-1, injected with *LvTRIM9-1* dsRNA and WSSV. Stars (**) indicate extremely significant differences (*p* < 0.01) of the gene expression levels between dsEGFP- and dsLvTRIM9-1-treated groups. All assays described above were biologically repeated for three times.

The NF-κB pathway, which could be regulated by TRIM proteins, is an essential pathway in regulation of shrimp antiviral immunity and viral propagation during WSSV infection. In order to know whether LvTRIM9-1 affected this signaling pathway, the expression level of the NF-κB transcription factor *LvRelish* in the lymphoid organ was detected in shrimp after *LvTRIM9-1* silencing. After knockdown of *LvTRIM9-1*, the expression levels of *LvRelish* (**Figure 3A**) were downregulated at all time points.

To confirm the role of LvTRIM9-1 in regulating the NF-κB pathway, dual-luciferase reporter assays were employed. Upon transfection with different levels of LvTRIM9-1 expression plasmids, the expression of LvTRIM9-1 gradually increased both at the mRNA level and at the protein level in HEK293T cells. The dual-luciferase reporter assay results showed that overexpression of LvTRIM9-1 protein could activate the NF-κB promoter activity, and this activation presented a concentration-dependent tendency (**Figure 3D**).

The NF-κB pathway affected viral propagation through regulating the expression of some WSSV genes and host AMP genes. Therefore, we detected the transcriptional changes of several WSSV genes in lymphoid organ and epidermis *via* qPCR. After knockdown of *LvTRIM9-1*, the expression levels of *wsv069* and *wsv303* were significantly downregulated in lymphoid organ at 24 and 48 hpi (**Figures 3B, C** **)**. However, the expression levels of *LvRelish* (**Figure 3E**), *wsv069*, and *wsv303* showed no significant difference in epidermis between the two groups (**Figures 3F, G** **)**. The viral early genes (*wsv079*, *wsv249*), structural proteins (*VP26*, *VP28*), and non-structural protein (*VP9*/*ICP11*) also showed similar expression profiles in the lymphoid organ and epidermis between the two groups (**Figure S1**). However, knockdown of *LvTRIM9-1* scarcely influenced the expression levels of AMP genes highly expressed in the lymphoid organ (**Figure S2**). The expression levels of all 12 detected genes showed no significant difference between the *LvTRIM9-1* knockdown group and the control group at 24 hpi. Nine of them showed no significant difference between the *LvTRIM9-1* knockdown group and the control group at 48 hpi. Only the expression levels of *Lvpenaeidin_2b* and *Lvpenaeidin_4a* were upregulated and the expression level of *LvALF6* was downregulated at 48 hpi in the lymphoid organ of the *LvTRIM9-1* knockdown group. Taken together, these results indicated that LvTRIM9-1, which was specifically expressed in the lymphoid organ, was a potent activator of the NF-κB pathway and this regulation affected the transcription of viral genes rather than host AMP genes in the lymphoid organ.

### LvTRIM9-1 Interacts With LvIMD Through Its SPRY Domain

**Figure 4:**
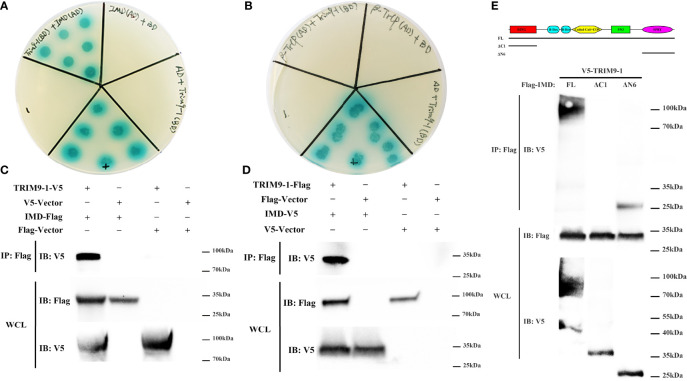
LvTRIM9-1 interacted with LvIMD through its SPRY domain. **(A, B)** Determination of the interaction between LvTRIM9-1 and LvIMD or Lvβ-TrCP by a Y2H assay. Y2H assay results showed that LvTRIM9-1 directly interacts with LvIMD but not with Lvβ-TrCP. The colonies were cultured on QDO/X/A plates (-Ade/-His/-Leu/-Trp quadruple dropout media with X-a-Gal and aureobasidin A). pGADT7-T and pGBKT7-p53 were used for the positive control (+); pGADT7-T and pGBKT7-Lam were used for the negative control (-). IMD(AD)+BD, β-TrCP(AD)+BD, and AD+TRIM9-1(BD) were used for the auto-activation detection. IMD(AD), pGADT7-LvIMD; β-TrCP(AD), pGADT7-Lvβ-TrCP; TRIM9-1(BD), pGBKT7-LvTRIM9-1; AD, pGADT7 vector; BD, pGBKT7 vector. **(C, D)** Determination of the interaction between LvTRIM9-1 and LvIMD by a Co-IP assay. Co-IP results showed that LvTRIM9-1 directly interacts with LvIMD. **(C)** Anti-V5 Western blot bands show the expression of LvTRIM9-1-V5, and anti-Flag Western blot bands show the expression of LvIMD-Flag. **(D)** Anti-Flag Western blot bands show the expression of LvTRIM9-1-Flag, and anti-V5 Western blot bands show the expression of LvIMD-V5. **(E)** Determination of the key region of LvTRIM9-1 involved in LvTRIM9-1 and LvIMD interaction by a Co-IP assay. The sites for mutation of LvTRIM9-1 are also labeled under the domain.

To determine the molecular mechanism how LvTRIM9-1 modulated the NF-κB pathway, we detected the interaction capability between LvTRIM9-1 and several candidate genes which could be ubiquitinated in the NF-κB pathway using the yeast two-hybrid assay. The results showed that LvTRIM9-1 could interact with LvIMD (**Figure 4A**), but not with Lvβ-TrCP (**Figure 4B**) or other detected proteins. Co-IP results also confirmed the interaction between LvTRIM9-1 and LvIMD (**Figures 4C, D** **)**.

To assess which domain of LvTRIM9-1 was responsible for the interaction with LvIMD, several LvTRIM9-1 deletion mutants were generated. Co-IP results showed that LvIMD could bind to wild-type LvTRIM9-1 (FL) and SPRY domain of LvTRIM9-1 (ΔN6), but not for the RING domain of LvTRIM9-1 (ΔC1) (**Figure 4E**). These data indicated that LvTRIM9-1 could interact with LvIMD through its SPRY domain.

## Discussion

TRIM proteins, a family of E3 ubiquitin ligase, are widely distributed in all metazoans (35). Compared to the high diversity of TRIM proteins in vertebrates, the number of TRIM proteins in invertebrates is generally low (36). In vertebrates, TRIM proteins are classified into eleven classes according to their various domains in the C-terminus, and class-I TRIM proteins contain six members comprising three pairs of paralogs, including TRIM1–TRIM18, TRIM9–TRIM67, and TRIM36–TRIM46 (14, 16). In invertebrates, TRIM9 is the only protein with the class-I motif architecture (26, 27). TRIM9 has only one copy in the genome of most species according to the data from Ensembl (release 104, May 2021), except limited fish species, probably attributed to genome duplication, including *Salmo salar*, *Carassius auratus*, and *Oncorhynchus mykiss* (**Table S3**) (37–39). The novel TRIM9, *LvTRIM9-1*, from *L. vannamei* showed a low sequence identity and different tissue distribution patterns compared with our previous reported *LvTRIM9* in *L*. *vannamei* (20). Phylogenetic analysis shows that LvTRIM9-1 and LvTRIM9 are clustered with Arthropoda TRIM9 proteins and belong to the invertebrate TRIM9 protein branch, which were separated from TRIM9, TRIM67, and other class-I TRIM proteins in Chordata. This evidence suggests that at least two copies of *TRIM9* genes exist in crustacean.

Although more than one copy of TRIM9 has already been reported in some fish species, their immune functions are not well illustrated. As important regulators of the innate immune system, it is interesting to clarify whether the two TRIM9 genes have different regulatory functions. We previously found that LvTRIM9 widely distributed in different tissues of shrimp and might be hijacked by WSSV for viral propagation through inhibiting the NF-κB pathway and downstream antimicrobial peptides production (20). The lymphoid organ-specific *LvTRIM9-1* was significantly responded to WSSV infection and exhibited positively effect on viral propagation in this tissue. At the early stage of WSSV infection, shrimp might attempt to inhibit the virus propagation *via* downregulation of *LvTRIM9-1*. With the infection process going on, the dose of WSSV increased dramatically and the virus might utilize its own or host’s regulators of the NF-κB pathway or ubiquitin system to promote its propagation (5, 40, 41). Therefore, we guess that WSSV might upregulate the expression of *LvTRM9-1*, which showed a recovery and boosting expression profiles from 6 to 24 hpi. However, the mechanism how WSSV controls the expression of *LvTRIM9-1* needs to be further investigated. Collectively, both LvTRIM9 and LvTRIM9-1 proteins seem to be beneficial for *in vivo* WSSV propagation.

Although both TRIM9 proteins in shrimp are beneficial for WSSV infection, their regulatory mechanisms are different. TRIM proteins usually act as ligases for ubiquitination (42). LvTRIM9 could directly interact with β-TrCP to negatively regulate the NF-κB pathway (20), which is very similar to the way that TRIM9 functions in human (8). In the present study, the expression of *LvRelish* as well as its regulated viral genes was downregulated upon knockdown of *LvTRIM9-1*, which indicated that *LvTRIM9-1* could regulate the NF-κB signaling pathway. Therefore, we surveyed all proteins that could be ubiquitinated in the mammalian NF-κB signaling pathway (43) and selected their homologs, including LvTAK1 (10), LvTRAF6 (44), LvIMD (6), LvGSK3β, and Lvβ-TrCP (20), in shrimp as candidates to screen their interaction with LvTRIM9-1. Unlike LvTRIM9 and TRIM9 proteins in human (18–20), yeast two-hybrid and Co-IP results revealed that LvTRIM9-1 could directly interact with LvIMD to activate the NF-κB signaling pathway. IMD, a death domain-containing protein homologous to mammalian RIP1, is a key adaptor protein and a major target of ubiquitination in the IMD pathway (6, 7). During this process, the SPRY domain of LvTRIM9-1 played an essential role in its interaction with LvIMD. In TRIM proteins, the SPRY domain is a key domain for their interactions with the substrates (42, 45) and approximately two-thirds of the TRIM proteins possess this domain (46). Upon stimulation, IMD can activate Relish to initiate the transcription of target genes (7, 8). Unlike *LvTRIM9*, knockdown of *LvTRIM9-1* had little effect on the expression of AMPs in shrimp but influenced the transcription of several immediate-early genes of WSSV. Among them, *wsv069* was the first identified immediate-early gene in WSSV, and its expression could be induced by the activation of host NF-κB pathway (4, 5). The activation of *wsv069* by shrimp NF-κB pathway can in turn induce viral genes by itself and establishes a positive-feedback loop to amplify the signaling and further activate other viral early and late genes (4). Consequently, other WSSV genes in the lymphoid organ were also downregulated by knockdown of *LvTRIM9-1*. These data suggested that LvTRIM9-1 could directly interact with LvIMD, the key component of the IMD pathway, through its SPRY domain and activate the IMD pathway to enhance the viral transcription.

It is worth noting that the regulatory function of LvTRIM9-1 on the NF-κB pathway seems to be restricted in the lymph organ. In shrimp, the lymphoid organ is a main immune organ with great phagocytic ability, which could filter and remove invading pathogens through bacteriostasis and viral degradation (47–49). In addition, the lymphoid organ-specific anti-lipopolysaccharide factors exhibit stronger antimicrobial activities against tested pathogenic bacteria than other ALFs, which suggested the importance of the lymphoid organ in shrimp humoral immunity (50, 51). However, the lymph organ exhibits extremely different immune responses against different pathogens. Several pattern recognition receptors, the proPO activating system, and phagocytosis-related genes were widely activated in the lymphoid organ after *Vibrio parahaemolyticus* challenge, whereas these processes were inhibited after WSSV infection, suggesting that the shrimp lymphoid organ plays different functions in response to the early infection of distinct pathogens (52). Collectively, although the lymphoid organ plays vital immune roles in shrimp, the virus could not only inhibit its immune responses but also utilize some immune pathways, like the LvTRIM9-1 regulated IMD pathway in this tissue to escape the host immune defense and promote its propagation.

**Figure 5:**
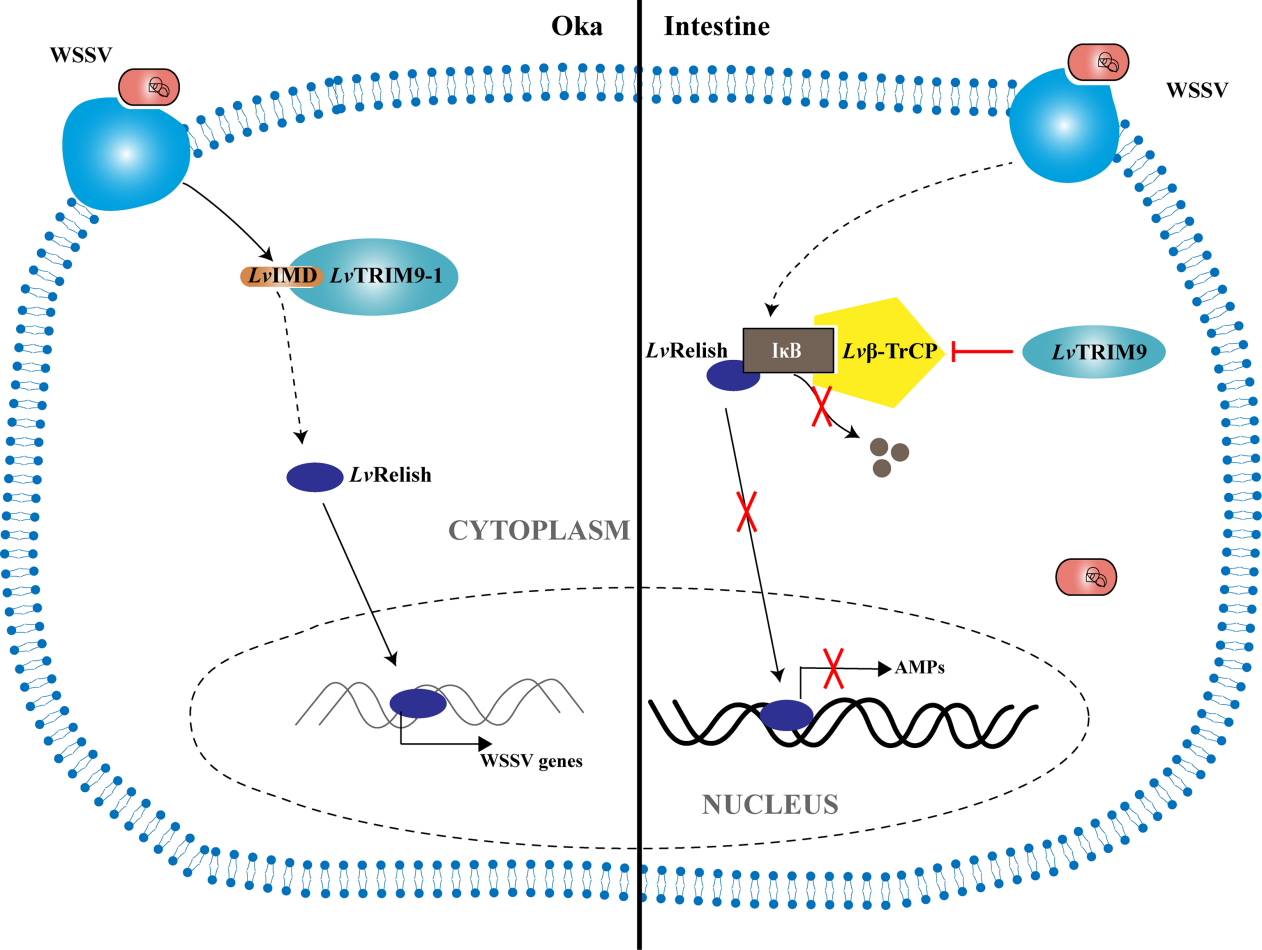
Model for the regulation of NF-κB signaling pathway during WSSV infection by LvTRIM9 and LvTRIM9-1. In the intestine, infection of host cells by WSSV leads to the activation of LvTRIM9, then the upregulated LvTRIM9 interacts with Lvβ-TrCP and inhibits the NF-κB pathway to decrease the production of downstream antimicrobial peptides including LvCrustinA, LvPEN3-1, Lvpenaeidin2b, and Lvpenaeidin4a, which results in WSSV propagation in shrimp. Although WSSV infection also results in the upregulation of LvTRIM9-1, LvTRIM9-1 modulates the NF-κB pathway-mediated responses in a different manner. Instead of interaction with Lvβ-TrCP, LvTRIM9-1 interacts with LvIMD *via* its SPRY domain and activates the NF-κB pathway, which leads to the activation of viral transcriptions including *wsv069* and *wsv303* for WSSV propagation.

In summary, a working model was proposed to illustrate how the shrimp TRIM9 genes, *LvTRIM9* and *LvTRIM9-1*, promote WSSV infection through distinct regulatory mechanisms (**Figure 5**). WSSV infection boosts the widely distributed LvTRIM9 and inhibits LvRelish-mediated AMP production in the intestine *via* interaction with Lvβ-TrCP. In contrast, the lymphoid organ-specific LvTRIM9-1 is activated by WSSV to bind to LvIMD *via* its SPRY domain and facilitates viral transcription through enhancing the IMD pathway. These results, together with previous reports on other TRIM proteins, collectively suggest the diverse roles of TRIM proteins in regulation of the invertebrate innate immunity.

## Data Availability Statement

The original contributions presented in the study are included in the article/**Supplementary Material**. Further inquiries can be directed to the corresponding authors.

## Author Contributions

SHL, JHX, and FHL supervised the overall project and designed the experiments. MZS performed the experiments, analyzed the data, and wrote the manuscript. SJJ and XCL helped to perform experiments and analyzed data. All authors reviewed the manuscript. All authors contributed to the article and approved the submitted version.

## Funding

This work was financially supported by the National Natural Science Foundation of China (31830100, 41776158, 31972829) and the China Agriculture Research System of MOF and MARA.

## Conflict of Interest

The authors declare that the research was conducted in the absence of any commercial or financial relationships that could be construed as a potential conflict of interest.

## Publisher’s Note

All claims expressed in this article are solely those of the authors and do not necessarily represent those of their affiliated organizations, or those of the publisher, the editors and the reviewers. Any product that may be evaluated in this article, or claim that may be made by its manufacturer, is not guaranteed or endorsed by the publisher.

## References

[1] Li CZ Wang S He JG. The Two NF-Kappa B Pathways Regulating Bacterial and WSSV Infection of Shrimp. Front Immunol (2019) 10:26. doi: 10.3389/fimmu.2019.01785 31417561 PMC6683665

[2] Li FH Xiang JH. Recent Advances in Researches on the Innate Immunity of Shrimp in China. Dev Comp Immunol (2013) 39:11–26. doi: 10.1016/j.dci.2012.03.016 22484214

[3] Li F Xiang J. Signaling Pathways Regulating Innate Immune Responses in Shrimp. Fish Shellfish Immun (2013) 34:973–80. doi: 10.1016/j.fsi.2012.08.023 22967763

[4] Huang X-D Zhao L Zhang H-Q Xu X-P Jia X-T Chen Y-H. Shrimp NF-κb Binds to the Immediate-Early Gene Ie1 Promoter of White Spot Syndrome Virus and Upregulates its Activity. Virology (2010) 406:176–80. doi: 10.1016/j.virol.2010.06.046 20684968

[5] Wang PH Gu ZH Wan DH Zhang MY Weng SP Yu XQ. The Shrimp NF-κb Pathway Is Activated by White Spot Syndrome Virus (WSSV) 449 to Facilitate the Expression of WSSV069 (Ie1), WSSV303 and WSSV371. PloS One (2011) 6:e24773. doi: 10.1371/journal.pone.0024773 21931849 PMC3171479

[6] Wang PH Gu ZH Huang XD Liu BD Deng XX Ai HS. An Immune Deficiency Homolog From the White Shrimp, Litopenaeus Vannamei, Activates Antimicrobial Peptide Genes. Mol Immunol (2009) 46:1897–904. doi: 10.1016/j.molimm.2009.01.005 19232438

[7] Kleino A Silverman N. The Drosophila IMD Pathway in the Activation of the Humoral Immune Response. Dev Comp Immunol (2014) 42:25–35. doi: 10.1016/j.dci.2013.05.014 23721820 PMC3808521

[8] Myllymäki H Valanne S Rämet M. The Drosophila Imd Signaling Pathway. J Immunol (2014) 192:3455–62. doi: 10.4049/jimmunol.1303309 24706930

[9] Huang XD Yin ZX Liao JX Wang PH Yang LS Ai HS. Identification and Functional Study of a Shrimp Relish Homologue. Fish Shellfish Immun (2009) 27:230–8. doi: 10.1016/j.fsi.2009.05.003 19463956

[10] Wang S Li H Lǚ K Qian Z Weng S He J. Identification and Characterization of Transforming Growth Factor β-Activated Kinase 1 From Litopenaeus Vannamei Involved in Anti-Bacterial Host Defense. Fish Shellfish Immun (2016) 52:278–88. doi: 10.1016/j.fsi.2016.03.149 27033469

[11] Wang S Li M Yin B Li H Xiao B Lu K. Shrimp TAB1 Interacts With TAK1 and P38 and Activates the Host Innate Immune Response to Bacterial Infection. Mol Immunol (2017) 88:10–9. doi: 10.1016/j.molimm.2017.05.016 28577391

[12] Wang PH Gu ZH Wan DH Liu BD Huang XD Weng SP. The Shrimp IKK–NF-κb Signaling Pathway Regulates Antimicrobial Peptide Expression and may be Subverted by White Spot Syndrome Virus to Facilitate Viral Gene Expression. Cell Mol Immunol (2013) 10:423–36. doi: 10.1038/cmi.2013.30 PMC3759962 23954949

[13] Wang P-H Wan D-H Gu Z-H Qiu W Chen Y-G Weng S-P. Analysis of Expression, Cellular Localization, and Function of Three Inhibitors of Apoptosis (IAPs) From Litopenaeus Vannamei During WSSV Infection and in Regulation of Antimicrobial Peptide Genes (AMPs). PloS One (2013) 8:e72592. doi: 10.1371/journal.pone.0072592 23967321 PMC3743791

[14] McNab FW Rajsbaum R Stoye JP O’Garra A. Tripartite-Motif Proteins and Innate Immune Regulation. Curr Opin Immunol (2011) 23:46–56. doi: 10.1016/j.coi.2010.10.021 21131187

[15] Hatakeyama S. TRIM Family Proteins: Roles in Autophagy, Immunity, and Carcinogenesis. Trends Biochem Sci (2017) 42:297–311. doi: 10.1016/j.tibs.2017.01.002 28118948

[16] Versteeg GA Benke S García-Sastre A Rajsbaum R. InTRIMsic Immunity: Positive and Negative Regulation of Immune Signaling by Tripartite Motif Proteins. Cytokine Growth F R (2014) 25:563–76. doi: 10.1016/j.cytogfr.2014.08.001 PMC7173094 25172371

[17] Kawai T Akira S. Regulation of Innate Immune Signalling Pathways by the Tripartite Motif (TRIM) Family Proteins. EMBO Mol Med (2011) 3:513–27. doi: 10.1002/emmm.201100160 PMC3377094 21826793

[18] Shi MD Cho H Inn KS Yang AR Zhao Z Liang QM. Negative Regulation of NF-Kappa B Activity by Brain-Specific TRIpartite Motif Protein 9. Nat Commun (2014) 5:4820. doi: 10.1038/ncomms5820 25190485 PMC4157316

[19] Qin YF Liu QX Tian S Xie WH Cui J Wang RF. TRIM9 Short Isoform Preferentially Promotes DNA and RNA Virus-Induced Production of Type I Interferon by Recruiting GSK3 Beta to TBK1. Cell Res (2016) 26:613–28. doi: 10.1038/cr.2016.27 PMC4856760 26915459

[20] Sun M Li S Yu K Xiang J Li F. An E3 Ubiquitin Ligase TRIM9 is Involved in WSSV Infection via Interaction With β-TrCP. Dev Comp Immunol (2019) 97:57–63. doi: 10.1016/j.dci.2019.03.014 30910419

[21] Zhao C Peng C Wang P Yan L Fan S Qiu L. Identification of a Shrimp E3 Ubiquitin Ligase TRIM50-Like Involved in Restricting White Spot Syndrome Virus Proliferation by Its Mediated Autophagy and Ubiquitination. Front Immunol (2021) 12:682562. doi: 10.3389/fimmu.2021.682562 34046043 PMC8144704

[22] Li W-D Chang X-J Zheng S-C Liu H-P. A Novel CQTRIM32 From Red Claw Crayfish Cherax Quadricarinatus Inhibits White Spot Syndrome Virus Infection. Fish Shellfish Immun (2019) 91:401–2. doi: 10.1016/j.fsi.2019.04.103

[23] Wang L Lu K-C Chen G-L Li M Zhang C-Z Chen Y-H. A Litopenaeus Vannamei TRIM32 Gene is Involved in Oxidative Stress Response and Innate Immunity. Fish Shellfish Immun (2020) 107:547–55. doi: 10.1016/j.fsi.2020.11.002 33161091

[24] Zhang RD Dai XL Cao XY Zhang C Wang KQ Huang X. Trim23 Promotes WSSV Replication Though Negative Regulation of Antimicrobial Peptides Expression in Macrobrachium Nipponense. Mol Immunol (2020) 124:172–9. doi: 10.1016/j.molimm.2020.06.007 32585511

[25] Peng C Zhao C Wang P Yan L Fan S Qiu L. TRIM9 is Involved in Facilitating Vibrio Parahaemolyticus Infection by Inhibition of Relish Pathway in Penaeus Monodon. Mol Immunol (2021) 133:77–85. doi: 10.1016/j.molimm.2021.02.002 33636432

[26] Boyer NP Monkiewicz C Menon S Moy SS Gupton SL. Mammalian TRIM67 Functions in Brain Development and Behavior. Eneuro (2018) 5. doi: 10.1523/ENEURO.0186-18.2018 PMC6002264 29911180

[27] Short KM Cox TC. Subclassification of the RBCC/TRIM Superfamily Reveals a Novel Motif Necessary for Microtubule Binding. J Biol Chem (2006) 281:8970–80. doi: 10.1074/jbc.M512755200 16434393

[28] Rodriguez J Boulo V Mialhe E Bachere E. Characterisation of Shrimp Haemocytes and Plasma Components by Monoclonal Antibodies. J Cell Sci (1995) 108:1043–50. doi: 10.1242/jcs.108.3.1043 7622592

[29] Hasson KW Hasson J Aubert H Redman RM Lightner DV. A New RNA-Friendly Fixative for the Preservation of Penaeid Shrimp Samples for Virological Detection Using cDNA Genomic Probes. J Virol Methods (1997) 66:227–36. doi: 10.1016/s0166-0934(97)00066-9 9255734

[30] Sun Y Li F Xiang J. Analysis on the Dynamic Changes of the Amount of WSSV in Chinese Shrimp Fenneropenaeus Chinensis During Infection. Aquaculture (2013) 376:124–32. doi: 10.1016/j.aquaculture.2012.11.014

[31] Zhang X Yuan J Sun Y Li S Gao Y Yu Y. Penaeid Shrimp Genome Provides Insights Into Benthic Adaptation and Frequent Molting. Nat Commun (2019) 10:356. doi: 10.1038/s41467-018-08197-4 30664654 PMC6341167

[32] Wang ZW Li SH Li FH Xie SJ Xiang JH. Identification and Function Analysis of a Novel Vascular Endothelial Growth Factor, LvVEGF3, in the Pacific Whiteleg Shrimp Litopenaeus Vannamei. Dev Comp Immunol (2016) 63:111–20. doi: 10.1016/j.dci.2016.05.020 27241034

[33] Chang Y-S Liu W-J Lee C-C Chou T-L Lee Y-T Wu T-S. A 3D Model of the Membrane Protein Complex Formed by the White Spot Syndrome Virus Structural Proteins. PloS One (2010) 5:e10718. doi: 10.1371/journal.pone.0010718 20502662 PMC2873410

[34] Livak KJ Schmittgen TD. Analysis of Relative Gene Expression Data Using Real-Time Quantitative PCR and the 2–ΔΔct Method. Methods (2001) 25:402–8. doi: 10.1006/meth.2001.1262 11846609

[35] Ozato K Shin DM Chang TH Morse HC. TRIM Family Proteins and Their Emerging Roles in Innate Immunity. Nat Rev Immunol (2008) 8:849–60. doi: 10.1038/nri2413 PMC3433745 18836477

[36] Langevin C Levraud JP Boudinot P. Fish Antiviral Tripartite Motif (TRIM) Proteins. Fish Shellfish Immun (2019) 86:724–33. doi: 10.1016/j.fsi.2018.12.008 30550990

[37] Berthelot C Brunet F Chalopin D Juanchich A Bernard M Noël B. The Rainbow Trout Genome Provides Novel Insights Into Evolution After Whole-Genome Duplication in Vertebrates. Nat Commun (2014) 5:3657. doi: 10.1038/ncomms4657 24755649 PMC4071752

[38] Chen Z Omori Y Koren S Shirokiya T Kuroda T Miyamoto A. *De Novo* Assembly of the Goldfish (Carassius Auratus) Genome and the Evolution of Genes After Whole-Genome Duplication. Sci Adv (2019) 5:eaav0547. doi: 10.1126/sciadv.aav0547 31249862 PMC6594761

[39] Lien S Koop BF Sandve SR Miller JR Kent MP Nome T. The Atlantic Salmon Genome Provides Insights Into Rediploidization. Nature (2016) 533:200–5. doi: 10.1038/nature17164 PMC8127823 27088604

[40] Chen AJ Gao L Wang XW Zhao XF Wang JX. SUMO-Conjugating Enzyme E2 UBC9 Mediates Viral Immediate-Early Protein SUMOylation in Crayfish to Facilitate Reproduction of White Spot Syndrome Virus. J Virol (2013) 87:636–47. doi: 10.1128/JVI.01671-12 PMC3536383 23097446

[41] Wang Z Chua HK Gusti AARA He F Fenner B Manopo I. RING-H2 Protein WSSV249 From White Spot Syndrome Virus Sequesters a Shrimp Ubiquitin-Conjugating Enzyme, PvUbc, for Viral Pathogenesis. J Virol (2005) 79:8764–72. doi: 10.1128/jvi.79.14.8764-8772.2005 PMC1168725 15994770

[42] Esposito D Koliopoulos MG Rittinger K. Structural Determinants of TRIM Protein Function. Biochem Soc T (2017) 45:183–91. doi: 10.1042/BST20160325 28202672

[43] Won M Byun HS Park KA Hur GM. Post-Translational Control of NF-Kappa B Signaling by Ubiquitination. Arch Pharm Res (2016) 39:1075–84. doi: 10.1007/s12272-016-0772-2 27287455

[44] Wang PH Wan DH Gu ZH Deng XX Weng SP Yu XQ. Litopenaeus Vannamei Tumor Necrosis Factor Receptor-Associated Factor 6 (TRAF6) Responds to Vibrio Alginolyticus and White Spot Syndrome Virus (WSSV) Infection and Activates Antimicrobial Peptide Genes. Dev Comp Immunol (2011) 35:105–14. doi: 10.1016/j.dci.2010.08.013 20816892

[45] James LC Keeble AH Khan Z Rhodes DA Trowsdale J. Structural Basis for PRYSPRY-Mediated Tripartite Motif (TRIM) Protein Function. P Natl Acad Sci (2007) 104:6200–5. doi: 10.1073/pnas.0609174104 PMC1851072 17400754

[46] Napolitano LM Meroni G. TRIM Family: Pleiotropy and Diversification Through Homomultimer and Heteromultimer Formation. IUBMB Life (2012) 64:64–71. doi: 10.1002/iub.580 22131136

[47] Duangsuwan P Phoungpetchara I Tinikul Y Poljaroen J Wanichanon C Sobhon P. Histological and Three Dimensional Organizations of Lymphoid Tubules in Normal Lymphoid Organ of Penaeus Monodon. Fish Shellfish Immun (2008) 24:426–35. doi: 10.1016/j.fsi.2007.12.011 18272398

[48] Rusaini Owens L. Insight Into the Lymphoid Organ of Penaeid Prawns: A Review. Fish Shellfish Immun (2010) 29:367–77. doi: 10.1016/j.fsi.2010.05.011 20580831

[49] Anggraeni MS Owens L. The Haemocytic Origin of Lymphoid Organ Spheroid Cells in the Penaeid Prawn Penaeus Monodon. Dis Aquat Organ (2000) 40:85–92. doi: 10.3354/dao040085 10782341

[50] Sun M Li S Lv X Xiang J Lu Y Li F. A Lymphoid Organ Specific Anti-Lipopolysaccharide Factor From Litopenaeus Vannamei Exhibits Strong Antimicrobial Activities. Mar Drugs (2021) 19:250. doi: 10.3390/md19050250 33925052 PMC8145222

[51] Li S Lv X Li F Xiang J. Characterization of a Lymphoid Organ Specific Anti-Lipopolysaccharide Factor From Shrimp Reveals Structure-Activity Relationship of the LPS-Binding Domain. Front Immunol (2019) 10:872:872. doi: 10.3389/fimmu.2019.00872 31110504 PMC6499195

[52] Wang F Li S Li F. Different Immune Responses of the Lymphoid Organ in Shrimp at Early Challenge Stage of Vibrio Parahaemolyticus and WSSV. Animals (2021) 11:2160. doi: 10.3390/ani11082160 34438618 PMC8388422

