# Supplementary material for: A Novel TRIM9 Protein Promotes NF-κB Activation Through Interacting With LvIMD in Shrimp During WSSV Infection
Source: Front Immunol. 2022 Feb 23;13:819881. doi: 10.3389/fimmu.2022.819881 (PMC8904877; doi:10.3389/fimmu.2022.819881)
Supplement: Supplementary file 1 [file DataSheet_1.docx]

Supplementary Material

**Table S1.** Primer sequences and corresponding annealing temperature of genes used in this study.

| **Name** | **Sequence (5’-3’)** | |
| --- | --- | --- |
| **cDNA cloning** | | |
| LvTRIM9-1-1F | | ATGGAGGAGGAAATACGGTGTCCCG |
| LvTRIM9-1-1R | | TGCGGCTACAGGTGCGTGCT |
| ***In situ* hybridization** | | |
| LvTRIM9-1-pF1 | | TAATACGACTCACTATAGGGCTGCCCCGCTACACTGCCAT |
| LvTRIM9-1-pR1 | | CTCTGTCTTGAAAGGTCTTGAGGTT |
| LvTRIM9-1-pF2 | | CTGCCCCGCTACACTGCCAT |
| LvTRIM9-1-pR2 | | TAATACGACTCACTATAGGGCTCTGTCTTGAAAGGTCTTGAGGTT |
| **RT-PCR and qPCR** | | |
| 18S-qF | | TATACGCTAGTGGAGCTGGAA |
| 18S-qR | | GGGGAGGTAGTGACGAAAAAT |
| LvTRIM9-1-qF | | ACTGTTTAGTTTGTCGGTGGTC |
| LvTRIM9-1-qR | | TGTCTTGAAAGGTCTTGAGGTT |
| HsActin-qF | | CGTGCGTGACATTAAGGAGAA |
| HsActin-qR | | GGAAGGAAGGCTGGAAGAGT |
| LvRelish-qF | | TCTAACCAATCACCACAGCAC |
| LvRelsih-qR | | TGGTAAACTCAGTGTTCGGG |
| LvCrustinⅥ-qF | | AACCGACCCAACAGACCC |
| LvCrustinⅥ-qR | | CCACCGACGAAGTTACCG |
| LvCrustinⅡ a-2-qF | | TGGCCTCGATAAGTGTTGC |
| LvCrustinⅡ a-2-qR | | TCGGTCGTTCTTCAGATGGT |
| LvCrustinⅡ a-3-qF | | CGCAGGATAAAGACAAGGC |
| LvCrustinⅡ a-3-qR | | TCCAGGACCGATACCACC |
| LvPEN3-1-qF | | CGGGAGCAGCAAGAACG |
| LvPEN3-1-qR | | CGATACCCAGGCCACCA |
| Lvpenaeidin_2b-qF | | ACTTTCCGTCTCAGATGCTC |
| Lvpenaeidin_2b-qR | | AAGCCAGGTTTCCATTGTC |
| Lvpenaeidin_4a-qF | | CCCTTTACCCAAACCATCC |
| Lvpenaeidin_4a-qR | | TCCTCTGTGACAACAATCCC |
| LvALF2-qF | | CCATTGCGAACAAACTCAC |
| LvALF2-qR | | CACGCCCGATCTGCTAC |
| LvALF4-qF | | TTCCTACGGTGAATTGTGAGC |
| LvALF4-qR | | TCCTGCCATTGAAGTAAAGC |
| LvALF5-qF | | TCAGGAAAGTTTCAACGAC |
| LvALF5-qR | | GACAAGAGACAAGATGGCG |
| LvALF6-qF | | CTACTTCATCGGGTCCGTCAC |
| LvALF6-qR | | TGTGGTTTGGCTTCTTCCTC |
| LvALF7-qF | | GGACCTTCTGGAGAGTGA |
| LvALF7-qR | | GAAAAACAAAACACGGGA |
| LvALF8-qF | | TGACGAATCTGCGAACTCCA |
| LvALF8-qR | | CGCCATCTTTGACCAGGGAA |
| wsv069-qF | | ACAACAACAGACCCTACCCG |
| wsv069-qR | | AAATACGACATAGCACCTCCA |
| wsv303-qF | | CCAGGTTGAACACGATGAT |
| wsv303-qR | | ATGTCCACGGGATTTAGGC |
| wsv079-qF | | TACGGAAGAACCACACAGG |
| wsv079-qR | | TGATTACGATTCGGACACG |
| wsv249-qF | | AAGGGGTGTAATAAGGATGA |
| wsv249-qR | | ATTGTTGGCAGAATAGTTGG |
| VP9-qF | | TATGAAGAAGTGAGGAAGAC |
| VP9-qR | | TCCATTGAGGACAAAAGTAG |
| VP26-qF | | GTCATCTCTACCGTCACAGC |
| VP26-qR | | TTCGACATAGTATTTCCCTT |
| VP28-qF | | AAACCTCCGCATTCCTGTGA |
| VP28-qR | | TCCGCATCTTCTTCCTTCAT |
| **RNAi** | | |
| LvTRIM9-1-dsF | | TAATACGACTCACTATAGGGCTGCCCCGCTACACTGCCAT |
| LvTRIM9-1-dsR | | TAATACGACTCACTATAGGGCTCTGTCTTGAAAGGTCTTGAGGTT |
| EGFP-dsF | | TAATACGACTCACTATAGGGCAGTGCTTCAGCCGCTACCC |
| EGFP-dsR | | TAATACGACTCACTATAGGGAGTTCACCTTGATGCCGTTCTT |
| **Dual-luciferase reporter assays** | | |
| pCDNA3.1-TRIM9-1-F | | CTTGGTACCGAGCTCGGATCCATGGAGGAGGAAATACGGTG |
| pCDNA3.1-TRIM9-1-R | | TGTGCTGGATTCTGCAGAATTCCATGTATCCGACTGGCTGTCGT |
| **Yeast two-hybrid assay** | | |
| pGBK-TRIM9-1-F | | ATGGCCATGGAGGCCGAATTCATGGAGGAGGAAATACGGTG |
| pGBK-TRIM9-1-R | | CCGCTGCAGGTCGACGGATCCTCATGTATCCGACTGGCTGTG |
| pGAD-TAK1-F | | GCCATGGAGGCCAGTGAATTCATGCACATATCTGCCATGGA |
| pGAD-TAK1-R | | CAGCTCGAGCTCGATGGATCCGAGGGGGGTCAGGGAGAG |
| pGAD-TRAF6-F | | GCCATGGAGGCCAGTGAATTCATGGAGAGTGTCGAAGAGTC |
| pGAD-TRAF6-R | | CAGCTCGAGCTCGATGGATCCCACACAGCTTTGCTTCTCTA |
| pGAD-IMD-F | | GCCATGGAGGCCAGTGAATTCATGGATAATATTAAGACAGATTCG |
| pGAD-IMD-R | | CAGCTCGAGCTCGATGGATCCAGGTGTAAGCTTCTTCAGCA |
| pGAD-GSK3β-F | | GCCATGGAGGCCAGTGAATTCATGAGTGGACGACCCAGGA |
| pGAD-GSK3β-R | | CAGCTCGAGCTCGATGGATCCTCAATTATCATTTACAGCAGCA |
| pGAD-β-TrCP-F | | GCCATGGAGGCCAGTGAATTCATGGACACTGAACCACTTTTGG |
| pGAD-β-TrCP-R | | CAGCTCGAGCTCGATGGATCCTTAGCTTCTGTCGCAGGTGG |
| **Co-Immunoprecipitation** | | |
| pDHsp70-TRIM9-1-F | | CTTGGTACCGAGCTCGGATCCATGGAGGAGGAAATACGGTG |
| pDHsp70-TRIM9-1-R | | TGCTGGATATCTGCAGAATTCCATGTATCCGACTGGCTGTCGT |
| pDHsp70-IMD-F | | CTTGGTACCGAGCTCGGATCCATGGATAATATTAAGACAGATTCG |
| pDHsp70-IMD-R | | TGCTGGATATCTGCAGAATTCCAAGGTGTAAGCTTCTTCAGCA |
| pDHsp70-TRIM9-1ΔC1-F | | GCACAGTGGCGGCCGCTCGAGATGGAGGAGGAAATACGGTG |
| pDHsp70-TRIM9-1ΔC1-R | | CCGCGGGCCCTCTAGACTCGAGCGCGCGGGGGTTCCTGT |
| pDHsp70-TRIM9-1ΔN6-F | | GCACAGTGGCGGCCGCTCGAGATGGAGTGCCGGCTGATC |
| pDHsp70-TRIM9-1ΔN6-R | | CCGCGGGCCCTCTAGACTCGAGCGTGTATCCGACTGGCTGTCGT |

**Table S2.** The information of TRIM proteins used in the present study.

| Species | Gene name | UniprotKB Accession number |
| --- | --- | --- |
| *Homo sapiens* | TRIM9 | Q9C026 |
| *Mus musculus* | TRIM9 | Q8C7M3-3 |
| *Danio rerio* | TRIM9 | Q6NZX0 |
| *Xenopus tropicalis* | TRIM9 | F7E188 |
| *Macaca mulatta* | TRIM9 | A0A1D5Q2P9 |
| *Rattus norvegicus* | TRIM9 | Q91ZY8 |
| *Gallus gallus* | TRIM9 | A0A1D5PZ60 |
| *Drosophila melanogaster* | TRIM9 | M9MRI4 |
| *Caenorhabditis elegans* | MADD-2 | B1GRL4 |
| *Homo sapiens* | TRIM67 | Q6ZTA4 |
| *Mus musculus* | TRIM67 | Q505D9 |
| *Xenopus tropicalis* | TRIM67 | F6U581 |
| *Macaca mulatta* | TRIM67 | F6S2G3 |
| *Rattus norvegicus* | TRIM67 | D3ZTX1 |
| *Xenopus laevis* | TRIM9 | A0A1L8F061 |
| *Xenopus laevis* | TRIM67 | A0A1L8G7R8 |
| *Homo sapiens* | TRIM18 | O15344 |
| *Homo sapiens* | TRIM1 | Q9UJV3 |
| *Mus musculus* | TRIM18 | O70583 |
| *Mus musculus* | TRIM1 | Q9QUS6 |
| *Danio rerio* | TRIM18 | E7FAU0 |
| *Xenopus tropicalis* | TRIM18 | Q6DEU6 |
| *Gallus gallus* | TRIM18 | Q90WD1 |
| *Rattus norvegicus* | TRIM1 | A0A0G2JWJ3 |
| *Rattus norvegicus* | TRIM18 | P82458 |
| *Xenopus laevis* | TRIM1 | D8WX03 |
| *Hydra vulgaris* | TRIM9 | T2M630 |
| *Trichinella spiralis* | TRIM9 | A0A0V1B9S1 |
| *Trachymyrmex septentrionalis* | TRIM9 | A0A195FV05 |
| *Sus scrofa* | TRIM18 | K7GSV3 |
| *Ficedula albicollis* | TRIM9 | U3JGS8 |
| *Ficedula albicollis* | TRIM18 | U3JPI3 |
| *Ficedula albicollis* | TRIM1 | U3KBH9 |
| *Canis lupus familiaris* | TRIM1 | E2RAN1 |
| *Canis lupus familiaris* | TRIM9 | E2RKC8 |
| *Canis lupus familiaris* | TRIM67 | F6V1Y9 |
| *Canis lupus familiaris* | TRIM18 | E2R7N3 |
| *Salmo salar* | TRIM9 | A0A1S3SS87 |
| *Salmo salar* | TRIM67 | A0A1S3S8H3 |
| *Salmo salar* | TRIM18 | A0A1S3NWR8 |
| *Salmo salar* | TRIM1 | A0A1S3RQU3 |
| *Homo sapiens* | TRIM36 | Q9NQ86 |
| *Homo sapiens* | TRIM46 | Q7Z4K8 |
| *Mus musculus* | TRIM46 | Q7TNM2 |
| *Mus musculus* | TRIM36 | Q80WG7 |
| *Danio rerio* | TRIM36 | F1QUB4 |
| *Danio rerio* | TRIM46b | Q1L8B2 |
| *Danio rerio* | TRIM46a | E7F8R6 |
| *Xenopus tropicalis* | TRIM36 | F7A2I4 |
| *Gallus gallus* | TRIM36 | F1NE64 |
| *Rattus norvegicus* | TRIM46 | A0A0G2JXN2 |
| *Rattus norvegicus* | TRIM36 | A0A0G2JW11 |
| *Xenopus laevis* | TRIM36 | Q6NU77 |
| *Sus scrofa* | TRIM36 | F1RLE9 |
| *Sus scrofa* | TRIM46 | F1RGR8 |
| *Ficedula albicollis* | TRIM36 | U3JIW9 |
| *Ficedula albicollis* | TRIM46 | U3JJW0 |
| *Canis lupus familiaris* | TRIM36 | E2QZT5 |
| *Canis lupus familiaris* | TRIM46 | F6V5Z8 |
| *Gallus gallus* | TRIM67 | XP_015139864.1 |
| *Crassostrea gigas* | TRIM9 | EKC37550.1 |
| *Xenopus tropicalis* | TRIM1 | XP_017952420.1 |
| *Gallus gallus* | TRIM1 | XP_015133862.2 |
| *Aplysia californica* | TRIM9 | XP_005099658.1 |
| *Alligator mississippiensis* | TRIM67 | XP_014452196.1 |
| *Alligator mississippiensis* | TRIM18 | XP_014463268.1 |
| *Alligator mississippiensis* | TRIM1 | XP_014465414.1 |
| *Alligator mississippiensis* | TRIM9 | XP_019349655.1 |
| *Ficedula albicollis* | TRIM67 | XP_005043498.1 |
| *Exaiptasia diaphana* | TRIM9 | KXJ14478.1 |
| *Helobdella robusta* | HELRODRAFT_117052 | XP_009012087.1 |
| *Opisthorchis viverrini* | T265_12702 | XP_009163316.1 |
| *Opisthorchis viverrini* | T265_00552 | XP_009162620.1 |
| *Saccoglossus kowalevskii* | TRIM9 | XP_002741117.1 |
| *Saccoglossus kowalevskii* | TRIM1 | XP_006823565.1 |
| *Acanthaster planci* | TRIM9 | XP_022109997.1 |
| *Litopenaeus_vannamei* | TRIM9 | QIX02857.1 |
| *Litopenaeus_vannamei* | TRIM9-1 | OK188769 |
| *Armadillidium nasatum* | TRIM9, partial | KAB7498956.1 |
| *Daphnia magna* | TRIM9 | JAM82903.1 |

**Table S3.** Number of TRIM9 proteins among several animal genomes.

| Species (alphabetic order) | Latin name | Numbers of TRIM9 protein | Note |
| --- | --- | --- | --- |
| Abingdon island giant tortoise | *Chelonoidis abingdonii* | 1 |  |
| African green monkey | *Chlorocebus sabaeus* | 1 |  |
| African ostrich | *Struthio camelus australis* | 1 |  |
| African savanna elephant | *Loxodonta africana* | 1 |  |
| Agassiz's desert tortoise | *Gopherus agassizii* | 1 |  |
| Algerian mouse | *Mus spretus* | 1 |  |
| Alpaca | *Vicugna pacos* | 1 |  |
| Amazon molly | *Poecilia formosa* | 1 |  |
| American beaver | *Castor canadensis* | 1 |  |
| American bison | *Bison bison bison* | 1 |  |
| American black bear | *Ursus americanus* | 1 |  |
| American mink | *Neovison vison* | 1 |  |
| American pika | *Ochotona princeps* | 1 |  |
| Angola colobus | *Colobus angolensis palliatus* | 1 |  |
| Arabian camel | *Camelus dromedarius* | 1 |  |
| Arctic ground squirrel | *Urocitellus parryii* | 1 |  |
| Argentine black and white tegu | *Salvator merianae* | 1 |  |
| Asian bonytongue | *Scleropages formosus* | 1 |  |
| Atlantic cod | *Gadus morhua* | 1 |  |
| Atlantic herring | *Clupea harengus* | 1 |  |
| Atlantic salmon | *Salmo salar* | 2 |  |
| Australian saltwater crocodile | *Crocodylus porosus* | 1 |  |
| Ballan wrasse | *Labrus bergylta* | 1 |  |
| Barramundi perch | *Lates calcarifer* | 1 |  |
| Beluga whale | *Delphinapterus leucas* | 1 |  |
| Bengalese finch | *Lonchura striata domestica* | 1 |  |
| Bicolor damselfish | *Stegastes partitus* | 1 |  |
| Black snub-nosed monkey | *Rhinopithecus bieti* | 1 |  |
| Blind barbel | *Sinocyclocheilus anshuiensis* | 4^a^ | ^a^ 4 are novel genes, TRIM9-like |
| Blue tit | *Cyanistes caeruleus* | 1 |  |
| Blue whale | *Balaenoptera musculus* | 1 |  |
| Blue-crowned manakin | *Lepidothrix coronata* | 1 |  |
| Blunt-snouted clingfish | *Gouania willdenowi* | 1 |  |
| Bolivian squirrel monkey | *Saimiri boliviensis boliviensis* | 1 |  |
| Bonobo | *Pan paniscus* | 1 |  |
| Bornean orangutan | *Pongo abelii* | 1 |  |
| Bottlenosed dolphin | *Tursiops truncatus* | 1 |  |
| Brown trout | *Salmo trutta* | 2 |  |
| Budgie | *Melopsittacus undulatus* | 1 |  |
| Burton's mouthbrooder | *Haplochromis burtoni* | 1 |  |
| California sea lion | *Zalophus californianus* | 1 |  |
| Cape rock hyrax | *Procavia capensis* | 1 |  |
| Cattle | *Bos taurus* | 1 |  |
| Central bearded dragon | *Pogona vitticeps* | 1 |  |
| Chacoan peccary | *Catagonus wagneri* | 1 |  |
| Channel bull blenny | *Cottoperca gobio* | 1 |  |
| Channel catfish | *Ictalurus punctatus* | 1 |  |
| Chicken | *Gallus gallus* | 1 |  |
| Chilean tinamou | *Nothoprocta perdicaria* | 1 |  |
| Chimpanzee | *Pan troglodytes* | 1 |  |
| Chinese hamster | *Cricetulus griseus* | 2^b^ | ^b^ 1 is novel gene, TRIM9-like |
| Chinese softshell turtle | *Pelodiscus sinensis* | 1 |  |
| Chinook salmon | *Oncorhynchus tshawytscha* | 2 |  |
| Climbing perch | *Anabas testudineus* | 1 |  |
| Clown anemonefish | *Amphiprion ocellaris* | 1 |  |
| Coelacanth | *Latimeria chalumnae* | 1 |  |
| Coho salmon | *Oncorhynchus kisutch* | 2 |  |
| Collared flycatcher | *Ficedula albicollis* | 1 |  |
| Common canary | *Serinus canaria* | 1 |  |
| Common carp | *Cyprinus carpio* | 1 |  |
| Common mallard | *Anas platyrhynchos platyrhynchos* | 1 |  |
| Common wall lizard | *Podarcis muralis* | 1 |  |
| Common wombat | *Vombatus ursinus* | 1 |  |
| Coquerel's sifaka | *Propithecus coquereli* | 1 |  |
| Crab-eating macaque | *Macaca fascicularis* | 1 |  |
| Damara mole rat | *Fukomys damarensis* | 1 |  |
| Degu | *Octodon degus* | 1 |  |
| Denticle herring | *Denticeps clupeoides* | 3 |  |
| Dingo | *Canis lupus dingo* | 1 |  |
| Dog | *Canis lupus familiaris* | 1 |  |
| Domestic cat | *Felis catus* | 1 |  |
| Domestic ferret | *Mustela putorius furo* | 1 |  |
| Domestic guinea pig | *Cavia porcellus* | 1 |  |
| Domestic yak | *Bos grunniens* | 1 |  |
| Donkey | *Equus asinus asinus* | 1 |  |
| Drill | *Mandrillus leucophaeus* | 1 |  |
| Eastern brown snake | *Pseudonaja textilis* | 1 |  |
| Eastern happy | *Astatotilapia calliptera* | 1 |  |
| Electric eel | *Electrophorus electricus* | 1 |  |
| Elephant shark | *Callorhinchus milii* | 1 |  |
| Emu | *Dromaius novaehollandiae* | 1 |  |
| Eurasian red squirrel | *Sciurus vulgaris* | 1 |  |
| European seabass | *Dicentrarchus labrax* | 1 |  |
| European shrew | *Sorex araneus* | 1 |  |
| Fruit fly | *Drosophila melanogaster* | 1 |  |
| Fugu | *Takifugu rubripes* | 1 |  |
| Gelada | *Theropithecus gelada* | 1 |  |
| Giant panda | *Ailuropoda melanoleuca* | 1 |  |
| Gilthead seabream | *Sparus aurata* | 1 |  |
| Goat | *Capra hircus* | 1 |  |
| Golden eagle | *Aquila chrysaetos chrysaetos* | 1 |  |
| Golden Hamster | *Mesocricetus auratus* | 1 |  |
| Golden snub-nosed monkey | *Rhinopithecus roxellana* | 1 |  |
| Golden-line barbel | *Sinocyclocheilus grahami* | 2 |  |
| Goldfish | *Carassius auratus* | 2 |  |
| Goodes thornscrub tortoise | *Gopherus evgoodei* | 1 |  |
| Gray mouse lemur | *Microcebus murinus* | 1 |  |
| Gray short-tailed opossum | *Monodelphis domestica* | 1 |  |
| Great Tit | *Parus major* | 1 |  |
| Greater amberjack | *Seriola dumerili* | 1 |  |
| Greater bamboo lemur | *Prolemur simus* | 1 |  |
| Greater horseshoe bat | *Rhinolophus ferrumequinum* | 1 |  |
| Guppy | *Poecilia reticulata* | 1 |  |
| Helmeted guineafowl | *Numida meleagris* | 1 |  |
| Hoffmann's two-fingered sloth | *Choloepus hoffmanni* | 1 |  |
| Horned golden-line barbel | *Sinocyclocheilus rhinocerous* | 4 |  |
| Horse | *Equus caballus* | 1 |  |
| Huchen | *Hucho hucho* | 2 |  |
| Human | *Homo sapiens* | 1 |  |
| Hybrid cattle | *Bos indicus x Bos taurus* | 2^c^ | ^c^ 1 is novel gene, TRIM9-like |
| Indian cobra | *Naja naja* | 1 |  |
| Indian glassy fish | *Parambassis ranga* | 3^d^ | ^d^ 3 are novel genes, TRIM9-like |
| Indian medaka | *Oryzias melastigma* | 1 |  |
| Inshore hagfish | *Eptatretus burgeri* | 1 |  |
| Japanese medaka HdrR | *Oryzias latipes* | 1 |  |
| Japanese medaka HSOK | *Oryzias latipes* | 1 |  |
| Japanese quail | *Coturnix japonica* | 1 |  |
| Javanese ricefish | *Oryzias javanicus* | 1 |  |
| Jewelled blenny | *Salarias fasciatus* | 1 |  |
| Kakapo | *Strigops habroptila* | 1 |  |
| Koala | *Phascolarctos cinereus* | 1 |  |
| Large flying fox | *Pteropus vampyrus* | 1 |  |
| Large yellow croaker | *Larimichthys crocea* | 1 |  |
| Leishan spiny toad | *Leptobrachium leishanense* | 1 |  |
| Leopard | *Panthera pardus* | 1 |  |
| Lesser Egyptian jerboa | *Jaculus jaculus* | 1 |  |
| Lion | *Panthera leo* | 1 |  |
| Little brown bat | *Myotis lucifugus* | 1 |  |
| Live sharksucker | *Echeneis naucrates* | 1 |  |
| Long-tailed chinchilla | *Chinchilla lanigera* | 1 |  |
| Lumpfish | *Cyclopterus lumpus* | 1 |  |
| Lyretail cichlid | *Neolamprologus brichardi* | 1 |  |
| Ma's night monkey | *Aotus nancymaae* | 1 |  |
| Macaque | *Macaca mulatta* | 1 |  |
| Mainland tiger snake | *Notechis scutatus* | 1 |  |
| Makobe Island cichlid | *Pundamilia nyererei* | 1 |  |
| Mangrove rivulus | *Kryptolebias marmoratus* | 1 |  |
| Medium ground-finch | *Geospiza fortis* | 1 |  |
| Mexican tetra | *Astyanax mexicanus* | 1 |  |
| Midas cichlid | *Amphilophus citrinellus* | 1 |  |
| Mouse | *Mus musculus* | 1 |  |
| Mummichog | *Fundulus heteroclitus* | 1 |  |
| Naked mole-rat | *Heterocephalus glaber* | 1 |  |
| Narwhal | *Monodon monoceros* | 1 |  |
| New Caledonian crow | *Corvus moneduloides* | 1 |  |
| Nile tilapia | *Oreochromis niloticus* | 1 |  |
| Nine-banded armadillo | *Dasypus novemcinctus* | 1 |  |
| Northern American deer mouse | *Peromyscus maniculatus bairdii* | 1 |  |
| Northern pike | *Esox lucius* | 1 |  |
| Northern tree shrew | *Tupaia belangeri* | 1 |  |
| Northern white-cheeked gibbon | *Nomascus leucogenys* | 1 |  |
| Norway rat | *Rattus norvegicus* | 1 |  |
| Okarito brown kiwi | *Apteryx rowi* | 1 |  |
| Olive baboon | *Papio anubis* | 1 |  |
| Orange clownfish | *Amphiprion percula* | 1 |  |
| Orbiculate cardinalfish | *Sphaeramia orbicularis* | 1 |  |
| Ord's kangaroo rat | *Dipodomys ordii* | 1 |  |
| Pachon cavefish | *Astyanax mexicanus* | 1 |  |
| Paramormyrops kingsleyae | *Paramormyrops kingsleyae* | 1 |  |
| Philippine tarsier | *Carlito syrichta* | 1 |  |
| Pig | *Sus scrofa* | 1 |  |
| Pig-tailed macaque | *Macaca nemestrina* | 1 |  |
| Pike-perch | *Sander lucioperca* | 1 |  |
| Pinecone soldierfish | *Myripristis murdjan* | 1 |  |
| Pink-footed goose | *Anser brachyrhynchus* | 1 |  |
| Platypus | *Ornithorhynchus anatinus* | 1 |  |
| Polar bear | *Ursus maritimus* | 1 |  |
| Rabbit | *Oryctolagus cuniculus* | 1 |  |
| Rainbow trout | *Oncorhynchus mykiss* | 2 |  |
| Red fox | *Vulpes vulpes* | 1 |  |
| Red-bellied piranha | *Pygocentrus nattereri* | 1 |  |
| Reedfish | *Erpetoichthys calabaricus* | 1 |  |
| Ruff | *Calidris pugnax* | 1 |  |
| Ryukyu mouse | *Mus caroli* | 1 |  |
| Sailfin molly | *Poecilia latipinna* | 1 |  |
| Sheep | *Ovis aries* | 1 |  |
| Sheepshead minnow | *Cyprinodon variegatus* | 1 |  |
| Shortfin molly | *Poecilia mexicana* | 1 |  |
| Shrew mouse | *Mus pahari* | 1 |  |
| Siamese fighting fish | *Betta splendens* | 1 |  |
| Siberian musk deer | *Moschus moschiferus* | 1 |  |
| Small tree finch | *Camarhynchus parvulus* | 1 |  |
| Small-eared galago | *Otolemur garnettii* | 1 |  |
| Sooty mangabey | *Cercocebus atys* | 1 |  |
| Southern platyfish | *Xiphophorus maculatus* | 1 |  |
| Sperm whale | *Physeter catodon* | 1 |  |
| Spiny chromis | *Acanthochromis polyacanthus* | 1 |  |
| Spotted gar | *Lepisosteus oculatus* | 2 |  |
| Spotted green pufferfish | *Tetraodon nigroviridis* | 1 |  |
| Steppe mouse | *Mus spicilegus* | 1 |  |
| Swamp eel | *Monopterus albus* | 1 |  |
| Tammar wallaby | *Notamacropus eugenii* | 1 |  |
| Thirteen-lined ground squirrel | *Ictidomys tridecemlineatus* | 1 |  |
| Three-spined stickleback | *Gasterosteus aculeatus* | 1 |  |
| Tiger | *Panthera tigris altaica* | 1 |  |
| Tiger tail seahorse | *Hippocampus comes* | 1 |  |
| Tongue sole | *Cynoglossus semilaevis* | 1 |  |
| Tropical clawed frog | *Xenopus tropicalis* | 1 |  |
| Tuatara | *Sphenodon punctatus* | 1 |  |
| Turbot | *Scophthalmus maximus* | 1 |  |
| Turkey | *Meleagris gallopavo* | 1 |  |
| Turquoise killifish | *Nothobranchius furzeri* | 1 |  |
| Ugandan red Colobus | *Piliocolobus tephrosceles* | 1 |  |
| Upper Galilee mountains blind mole rat | *Nannospalax galili* | 1 |  |
| Vaquita | *Phocoena sinus* | 1 |  |
| Vole | *Microtus ochrogaster* | 1 |  |
| Western European hedgehog | *Erinaceus europaeus* | 1 |  |
| Western Lowland Gorilla | *Gorilla gorilla gorilla* | 1 |  |
| Western painted turtle | *Chrysemys picta bellii* | 1 |  |
| White-headed capuchin | *Cebus capucinus imitator* | 1 |  |
| White-tufted-ear marmoset | *Callithrix jacchus* | 1 |  |
| Wild yak | *Bos mutus* | 1 |  |
| Yarkand deer | *Cervus hanglu yarkandensis* | 1 |  |
| Yellowtail amberjack | *Seriola lalandi dorsalis* | 1 |  |
| Zebrafish | *Danio rerio* | 1 |  |
| Zebra mbuna | *Maylandia zebra* | 1 |  |
| Zig-zag eel | *Mastacembelus armatus* | 1 |  |


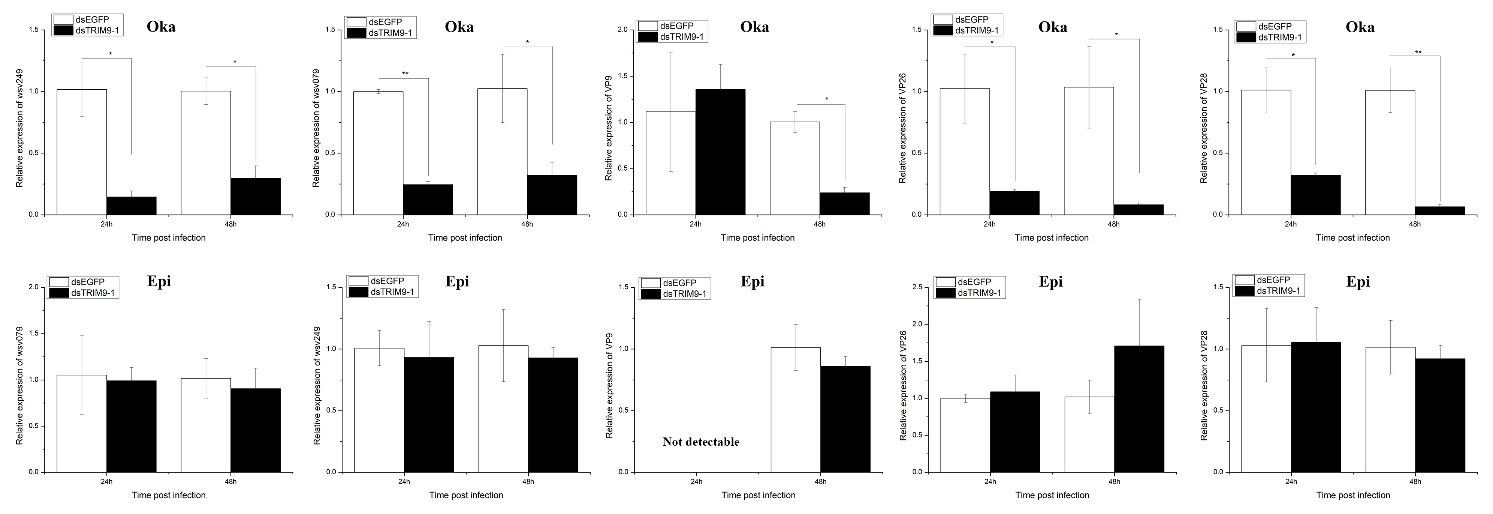


**FIGURE S1.** The mRNA expression levels of *wsv079*, *wsv303*, *VP9*, *VP26*, and *VP28* in lymphoid organ and epidermis at different hours after silencing of LvTRIM9-1 and WSSV infection. dsEGFP, injected with EGFP dsRNA and WSSV; dsTRIM9-1, injected with LvTRIM9-1 dsRNA and WSSV. Stars (**) indicate extremely significant differences (*P* < 0.01) of the gene expression levels between dsEGFP and dsLvTRIM9-1 treated groups. All assays described above were biologically repeated for three times.


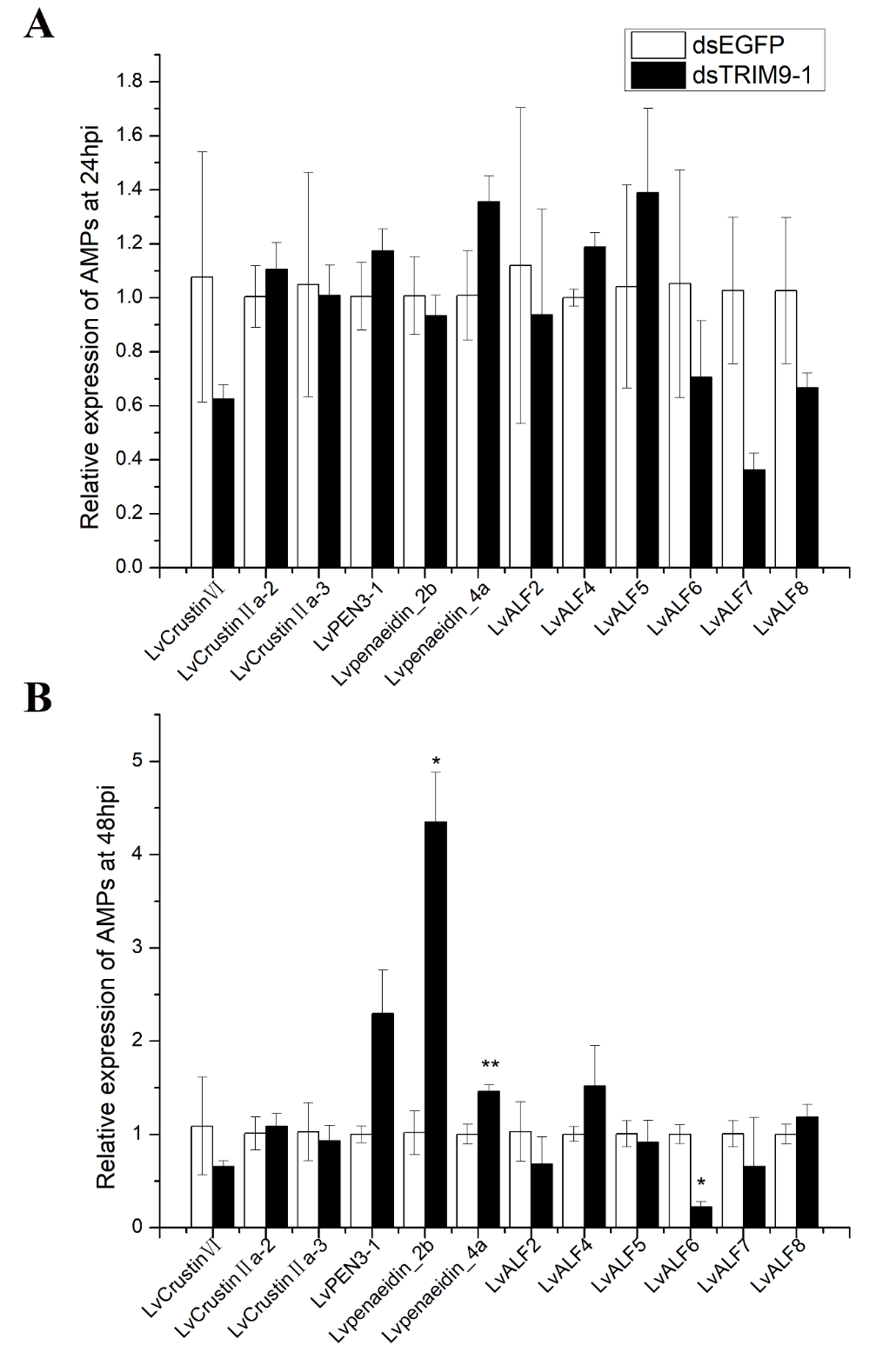


**Figure S2.** The mRNA expression levels of the AMP genes in lymphoid organ of LvTRIM9-1 knockdown shrimp at 24 hpi (**A**) and 48 hpi (**B**). The AMP genes of L. vannamei were obtained from GenBank (Crustin Ⅵ, GenBank accession number: KY351820; Crustin Ⅱ2-a, AY488493; Crustin Ⅱa-3, JQ824114; PEN3-1, DQ206403; Penaeidin 2b, AF390146; Penaeidin 4a_AF390147; ALF2, XM_027361790; ALF4, XM_027372864; ALF5, XM_027383848; ALF6, MF135542; ALF7, XM_027351129; ALF8, XM_027364058). All assays described above were biologically repeated for three times.
